# Coordinated optimization of reserve fund scale and emergency resource allocation for power utilities under multi-type large-scale blackout risks

**DOI:** 10.1371/journal.pone.0352511

**Published:** 2026-09-24

**Authors:** Yongju Gu, Zhiwei Zhang, Liyun Zhang

**Affiliations:** 1 School of Accounting, Chengdu College of Arts and Sciences, Chengdu, China; 2 School of Economics and Management, Southwest Petroleum University, Chengdu, China; 3 School of Science, Xihua University, Chengdu, China; University of Mazandaran, IRAN, ISLAMIC REPUBLIC OF

## Abstract

Large-scale blackouts increasingly arise from heterogeneous causes such as natural disasters, equipment failures and cyber attacks, which force power utilities to decide, before any event is known, both how large a financial reserve to hold and how to convert it into emergency response capability. This paper develops a coordinated optimization of reserve fund scale and emergency resource allocation under multi-type large-scale blackout risk. A unified set of multi-type blackout scenarios is constructed by Monte Carlo sampling and condensed by a tail-aware scenario reduction into a tractable representative set. The restoration technology distinguishes a repair channel, in which crews and materials enter as complementary inputs, from a re-energization channel supplied by mobile equipment, and both channels carry cause-dependent productivity so that the response mechanism differs across disasters, failures and cyber attacks. A two-stage risk-averse stochastic program co-optimizes the first-stage reserve fund and resource pre-positioning together with the second-stage post-event response, embeds a conditional-value-at-risk measure to limit extreme losses, and is solved by a sample-average-approximation scheme with an L-shaped decomposition. Case studies on a stylized 118-node planning instance show that the coordinated risk-averse plan attains an out-of-sample risk-adjusted cost of 352.1 $M against 558.2 $M for a decoupled scheme and 604.7 $M for a risk-neutral scheme, while holding CVaR_0.95_ to 154.3 $M and expected unserved energy to 1478 MWh. The tail-aware reduction keeps the CVaR_0.95_ error at 1.08% of its raw-sample value with 200 retained scenarios, against 11.91% for random subsampling. The cause mix governs the resource portfolio, and the value of lost load is the dominant economic driver of the appropriate reserve scale, with a standardized regression coefficient of 0.66 under joint parameter uncertainty. The study is presented as a methodological proof of concept: the instance is synthetic and network operability is not modelled, so the results characterize the structure of the preparedness trade-off rather than a validated prescription for a specific utility.

## Introduction

Modern society and economic activity depend on a continuous supply of electricity, which makes large-scale blackouts one of the most severe forms of infrastructure failure. Such events no longer originate from a single well-understood cause: extreme weather, the ageing and cascading failure of physical equipment, and the growing exposure of digitalized grids to cyber intrusion together constitute a spectrum of heterogeneous threats that power utilities must bear simultaneously [1, 2]. The economic stake is severe, because disruption propagates far beyond the electricity sector. It ripples through interdependent firms and supply chains and produces losses that greatly exceed the cost of the physical damage itself [3]. As outage events become more frequent and more diverse in origin, utilities are expected not only to restore service rapidly once an event has occurred [4], but also to prepare in advance for a wide and uncertain range of failure modes [5]. This dual task, anticipating multi-type blackout risks before they materialize and responding effectively afterwards, places emergency preparedness and its financial support at the centre of utility risk management.

One line of research characterizes blackout risk probabilistically and translates it into representative scenarios suitable for decision-making. Multi-hazard probabilistic risk assessment combines hazard occurrence, component fragility and economic loss into integrated risk metrics [6], while fragility- and vulnerability-based models describe how individual grid components fail under climatic and weather extremes such as hurricanes [7, 8]. Other studies frame extreme outages from an insurance and catastrophe-risk perspective [9], or quantify post-contingency vulnerability and recovery through statistical and probabilistic frameworks [10, 11]. These works have advanced the quantitative understanding of how disruptions arise and spread; most of them, however, concentrate on a single hazard category, usually weather, and devote little attention to constructing and managing a unified scenario set spanning natural, technical and cyber causes at once. The tension between the richness of the scenario set and computational tractability therefore remains unresolved, and it leaves open the question of how a large and heterogeneous population of blackout scenarios can be generated and then reduced to a manageable but representative subset.

A second and more mature line of work studies how physical emergency resources, namely repair crews, mobile power sources, spare equipment and the network reconfiguration they enable, should be positioned and dispatched to recover service after an outage. Many studies co-optimize the routing of repair crews together with mobile or transportable generators in order to accelerate restoration at the distribution and transmission level [12–15], and related models coordinate field teams for the energization of severely damaged systems [16]. Beyond purely reactive recovery, a complementary branch introduces pre-event preparedness and planning decisions, ranging from robust preparedness planning under extreme rainfall [17] and two-stage emergency-resource dispatch [18] to cooperative resilience-oriented planning of integrated energy systems [19]; comprehensive reviews confirm that resource allocation and restoration now form a well-developed core of distribution-system resilience research [20]. These models nonetheless treat the resource budget as exogenously fixed, and they optimize the deployment of personnel and equipment without asking how much financial reserve is needed to support them, or how that reserve should be sized in the first place.

The question of how much capital should be set aside, and how this financial commitment is converted into protective capability, has been studied from the financial and investment side. Resilience-oriented investment models decide where to harden the network or where to place microgrids under a limited budget [21, 22], and comparable preparedness studies in disaster operations optimize investment across inventory, capacity and capability so as to maximize the assistance a system can ultimately deliver [23, 24]. Parallel work in disaster risk finance examines how fiscal buffers, contingent instruments and insurance interact when a jurisdiction faces multiple correlated hazards [25], and humanitarian relief network design places facility location, inventory and allocation inside a single distributionally robust programme [26]. These works make clear that the level of financial commitment fundamentally shapes the protection that can be afforded. The funding decision, however, is usually modelled as an upstream budget constraint given to the resource problem, rather than as a reserve whose appropriate scale is itself an outcome of the downstream emergency response it must support. As a result, the scale of the financial reserve and the configuration of emergency resources are decided separately, which conceals the coupling that a reserve becomes useful only through the resources it funds, and that resources are deliverable only to the extent the reserve permits. Such an artificial separation risks both under-preparation and idle, redundant capacity.

From the methodological side, decisions that must be committed before uncertainty is resolved and adjusted afterwards are naturally formulated as two-stage or multi-stage stochastic programs, an approach widely applied to resilient energy systems and post-disruption logistics response [27–29]. Preparedness against rare, high-impact blackouts is driven by the tail of the loss distribution rather than by its mean, so a risk-averse formulation is essential, and the conditional value-at-risk (CVaR) has become the most common measure for embedding tail-loss control directly into such programs in virtual power plants, energy communities and integrated energy operations [27,30–33]. To keep these formulations tractable when the underlying scenario set is large, the sample average approximation (SAA) offers a principled way to approximate expected and risk-adjusted costs from a finite sample. Recent work has sharpened both ingredients: scenario reduction has been recast as an optimization problem with explicit quality guarantees on the reduced-set objective [34], decomposition schemes have been developed specifically for large risk-averse expansion planning [35], and the broader ambiguity-aware literature clarifies what a finite representative set can and cannot certify about the underlying distribution [36]. Surveys of power-system optimization place these developments in a longer methodological trajectory [37]. Although these techniques are individually well established, they are seldom combined to confront the joint challenge of a genuinely multi-type, high-cardinality scenario set together with a decision structure in which the financial reserve and the emergency resources are co-optimized.

Taken together, the reviewed literature leaves three specific gaps rather than a general absence of work. First, blackout-risk studies are organized around a single hazard category, so no construction exists that generates natural, technical and cyber outage scenarios within one probabilistic model and then reduces them under a criterion that is verifiably faithful in the upper tail, which is precisely the region a reserve exists to absorb; distance-based reduction alone does not certify tail fidelity, and the reduction literature that does provide guarantees has not been applied to heterogeneous multi-cause outage attributes. Second, the reserve scale is exogenous in every preparedness model reviewed above: the budget enters as a constraint parameter, so the marginal value of an additional unit of financing is never priced against the marginal loss it avoids, and the resulting plans cannot answer how large the cushion should be. Third, the restoration technology in existing multi-resource models converts heterogeneous resources into restored load through a single additive capability term, which makes crews, materials and equipment mutually substitutable and prevents the model from reproducing the cause-specific response mechanisms that motivate holding a diversified portfolio in the first place. These three gaps are complementary, and addressing any one of them in isolation leaves the coordination problem unresolved.

To address these gaps, this paper develops a coordinated optimization of reserve fund scale and emergency resource allocation for power utilities facing multi-type large-scale blackout risks. The methodological contribution is not the use of scenario reduction, CVaR or SAA individually, all of which are standard, but the specific way in which they are combined and adapted, and the contributions are stated at that level of specificity:

1) A unified multi-type blackout scenario construction is proposed in which disaster-, failure- and attack-induced outages are generated from one probabilistic model with cause-specific spatial signatures, and is condensed by a reduction whose distance metric is defined jointly over a categorical cause attribute and continuous damage attributes, with the normalization deliberately weighted towards severity so that the upper tail survives condensation. Tail fidelity is then established quantitatively, in terms of VaR, CVaR and extreme-quantile errors and of the out-of-sample regret of the resulting first-stage decision, against random subsampling, plain medoid clustering and fast-forward selection, rather than asserted from the visual agreement of cumulative distributions.
2) A two-stage risk-averse stochastic program is established in which the reserve fund scale is an endogenous first-stage variable co-optimized with resource pre-positioning, and in which the restoration technology separates a repair channel, where crews and materials are complementary inputs bound by a Leontief-type joint-input constraint, from a re-energization channel supplied by mobile equipment, with cause-dependent productivity and coverage in both channels. This is what allows the cause mix to reshape the resource portfolio endogenously instead of by assumption.
3) A solution and validation scheme is developed that separates the statistical errors induced by Monte Carlo sampling, scenario reduction, optimization and out-of-sample evaluation, constructs a lower-bound estimator on unreduced independent samples so that the reported optimality gap is statistically meaningful after reduction, and solves the resulting program by an L-shaped decomposition whose master problem, subproblems, cut coefficients and treatment of the CVaR variables are derived explicitly.

The remainder of this paper is organized as follows. The Problem description section sets out the problem of coordinating reserve funds and emergency resources under multi-type large-scale blackouts, including the decision structure and the construction and reduction of the multi-type scenario set. The Mathematical formulation section formulates the two-stage stochastic programming model, embeds the CVaR-based risk constraint, and presents the SAA-based solution method together with the decomposition algorithm. The Case studies section examines the model and analyses the sensitivity of key parameters. The Conclusion summarizes the findings and indicates directions for future work.

## Problem description

### Decision setting of reserve funding and emergency resources under blackout uncertainty

This section describes the decision problem a power utility faces when it must prepare for and respond to large-scale blackouts of heterogeneous origin. The utility considered here operates a transmission or distribution service territory whose continuity of supply is exposed to three broad categories of disruptive event: natural disasters such as typhoons, ice storms and floods; ageing-driven failure and cascading outage of physical equipment; and deliberate cyber attacks on an increasingly digitalized control and communication layer. Although these causes differ in physical mechanism, from the utility’s viewpoint they share a common consequence, namely a sudden loss of supply over a possibly wide area, together with large economic loss, recovery expenditure and social impact. The problem is therefore framed from the angle of corporate risk management: the utility must decide, long before any particular event is known, how much financial capacity to set aside and how much physical response capability to put in place, so that the consequences of a realized event can be contained at an acceptable cost.

The scope of the model is fixed at the outset, because several modelling choices below follow from it. The formulation is an aggregate strategic planning model for reserve sizing and depot-level pre-positioning over a preparedness horizon, not an operational dispatch model. Restoration is represented at the level of served load per unit of deployed resource, so no power-flow, voltage, line-capacity or islanding constraint enters; the network topology supplies the spatial layout of nodes, depots and critical loads, and the reachability structure, but not an electrical feasibility condition. Resource movement is represented by aggregate availability and cause-dependent reachability rather than by origin–destination flows, vehicle routing, congestion or return trips. Claims made in this paper are correspondingly confined to the sizing and composition of the reserve and of the pre-positioned portfolio, and to the way those quantities respond to risk preference, cause mix and economic parameters; they do not extend to detailed spatial allocation or to operational restoration schedules, for which the routing-based models cited above remain the appropriate instrument.

The financial instrument at the centre of this problem is a dedicated reserve fund, which is distinct from the resource-allocation decisions the literature has studied separately. The reserve is the pool of readily deployable capital that the utility maintains specifically for emergency preparedness and response, and not the capital used for routine operations or long-horizon network reinforcement. Its scale determines both how much physical capability can be staged before an event and how much emergency procurement can be afforded after one, and it also expresses the broader financial posture that a utility adopts towards catastrophic risk. Alternative mechanisms such as catastrophe insurance and contingent credit are not interchangeable with a cash reserve: they differ materially in trigger conditions, payout timing, coverage limits, collateral requirements, counterparty risk and basis risk, and a model that prices all of them through a single holding rate cannot represent those differences. The present formulation therefore does not claim to compare funding instruments. It represents one instrument, a liquid pre-event reserve, and treats ρ as the opportunity cost of committing capital to that instrument; the wider portfolio question of how cash, insurance and contingent credit should be combined is identified in the Conclusion as an extension rather than as a result of this work [9, 25]. Treating the reserve as an endogenous decision rather than a fixed budget is what allows the model to ask the question that motivates this work: how large the cushion should be, given the resources it will ultimately have to purchase and stage.

**Fig 1:**
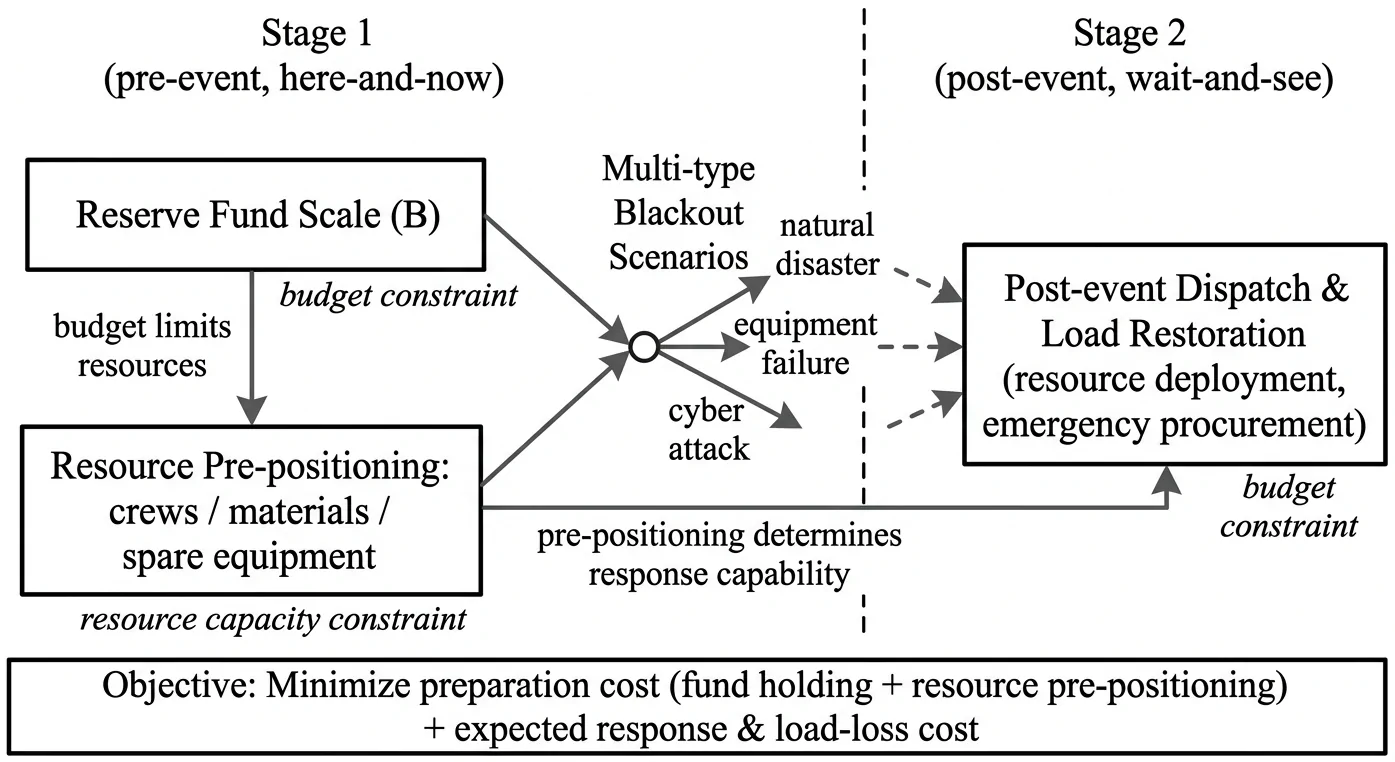
Two-stage decision framework of the preparedness problem. Coupling of the reserve fund scale, emergency resource pre-positioning, and post-event dispatch under multi-type blackout scenarios.

A defining feature of this setting is that decisions occur on two distinct time layers, which gives the problem its two-stage structure. In the first stage, before any blackout event is realized, the utility commits to a reserve fund scale and to the pre-positioning of emergency resources. These commitments are made under uncertainty and cannot be revised once an event strikes; together they constitute the preparedness posture of the utility. In the second stage, after a specific blackout scenario has occurred, the utility responds within the envelope of what it has prepared: it dispatches the pre-positioned resources, restores load according to operational priorities, and, when necessary and affordable, procures additional resources on an emergency basis. First-stage decisions therefore have a here-and-now character, while second-stage decisions are recourse actions adapted to the observed scenario. This separation between irreversible preparation and scenario-dependent response is the backbone of the problem, is illustrated in Fig 1, and places the problem within the established framework of two-stage stochastic programming for power-system resilience [38].

The emergency resources that the utility configures are deliberately treated as a heterogeneous portfolio rather than a single aggregate quantity, because the realism of the coordination problem depends on this distinction. Three representative classes are distinguished: repair crews, whose availability governs the speed at which damaged components can be restored; emergency materials and consumables such as poles, conductors and fuel, which are consumed during restoration; and spare or backup equipment such as mobile transformers and mobile emergency generators, whose deployment can re-energize critical load directly. Each class carries its own pre-positioning cost, dispatch cost, restoration capability and practical limit on how much can be staged in advance. The reserve fund, for its part, is not a free resource: holding capital in liquid and readily deployable form entails an opportunity cost, so the fund scale cannot be enlarged arbitrarily without penalty.

The three resource classes do not act in isolation but interact through both complementarity and substitution, and capturing this interaction is part of what makes the configuration problem non-trivial. Restoring a damaged feeder normally requires crews and materials together: skilled labour is of little use without the hardware it installs, and stocked hardware sits idle without crews to deploy it, so the two are strongly complementary, and a reserve that funds one without the other is poorly spent. Spare equipment such as mobile generators plays a different role: it can re-energize critical load directly and partially substitutes for the slower process of physical repair, and it is especially valuable in the early hours of an outage or in scenarios where access constraints delay conventional restoration. A sound preparedness plan therefore cannot size each resource class independently against an average need; it must balance the portfolio so that complementary resources are staged in compatible proportions and substitutable resources are held where they can shorten the restoration of the most valuable load. The Mathematical formulation section encodes this distinction explicitly, through a joint-input constraint that binds crew effort to material consumption within the repair channel and a separate re-energization channel for mobile equipment, rather than through a single additive capability term that would render the three classes mutually substitutable.

Equally important is the temporal dimension of the response, because the harm caused by a blackout is not a single lump sum but accumulates with the duration of the interruption. Critical loads that can tolerate a short outage become progressively more costly to leave unserved as hours pass, so the marginal value of restoration is highest in the early periods and for the most sensitive customers. Recourse is therefore a sequence of decisions over a restoration horizon rather than a one-shot allocation: crews and equipment are deployed, released and redeployed across periods, materials are consumed as work proceeds, and emergency procurement may arrive with a delay. Recognizing this temporal structure matters for sizing the reserve and the resources, because a configuration that restores the same total energy more quickly avoids the largest early-period losses. The model of the next section therefore resolves the second stage period by period and lets the value of speed be weighed against its cost.

The heterogeneity of the three cause types drives the preparedness decision substantially, which is why a unified treatment is required. Natural disasters tend to produce spatially correlated wide-area damage whose footprint follows the path of the hazard, so that many nodes fail together and restoration competes for a limited pool of crews and materials across a large region simultaneously; the binding constraint in such scenarios is usually the sheer volume of physical repair work. Equipment failures are typically more localized and are driven by asset age, loading history and maintenance backlog, so their scenarios affect fewer nodes but may concentrate on critical components whose loss carries disproportionate consequences. Cyber attacks belong to a different regime again: they may disable monitoring and control functions without immediate physical destruction, and produce outages that are geographically dispersed, difficult to diagnose, and dependent on backup equipment and manual operation rather than on conventional repair. The cyber class is represented here at the level of its outage consequence, that is, a dispersed footprint whose restoration relies on re-energization rather than repair, and not at the level of the cyber-physical mechanism. Loss of observability, communication degradation, data-integrity compromise, operator response and restoration coordination are not modelled, and the conclusions drawn for this class therefore concern a synthetic outage regime with those consequence characteristics rather than cyber resilience in general [39]. Because the scarce resource, the attainable restoration speed and the value of holding particular equipment all differ across these regimes, a preparedness plan tuned to one cause can be markedly inefficient against another. Treating the three jointly within a single scenario set is therefore essential for sizing a reserve and a resource portfolio that are robust across the full spectrum of threats rather than optimized for a single dominant hazard.

From these elements, the objective and the constraint system of the problem follow naturally. The utility seeks to minimize the total expected cost of preparedness and response, comprising the first-stage preparation cost, that is the opportunity cost of the reserve fund together with the cost of pre-positioning resources, and the second-stage cost incurred across blackout scenarios, which aggregates resource dispatch cost, emergency procurement cost, and the value of load that cannot be restored. The constraints follow from physical and financial reality: total pre-positioning expenditure cannot exceed the reserve fund; the quantity of each resource staged in advance is bounded by depot and logistical capacity; the response in any scenario is limited by the resources actually available, by the restoration capability of those resources, and by the load physically at risk; and restoration must respect geographic reach and the time window within which it remains useful. The economic tension carried by the objective explains why the problem cannot be resolved by intuition alone. If the reserve fund and the pre-positioned resources are set too low, the utility will be unable to respond when a severe event strikes, load remains unserved, and the value-of-lost-load penalty, together with the social and regulatory consequences it represents, dominates the outcome. If they are set too high, capital is tied up and equipment is staged but idle in the many scenarios where it is not needed, incurring holding and pre-positioning costs that are never recovered. The appropriate level lies between these extremes and depends on the full distribution of blackout outcomes rather than on any single representative event; it is exactly this balance, struck under multi-type uncertainty and with explicit attention to the tail, that the model is built to find.

The central difficulty motivating the modelling work of the next section is the intrinsic coupling between financial and physical decisions under deep uncertainty. The reserve fund scale sets the ceiling on the resources that can be pre-positioned, while the pre-positioned resources determine response capability and hence the losses incurred once a scenario unfolds; the two are linked through the two-stage structure and are not separable problems. At the same time, the consequences of multi-type blackouts are highly uncertain and dominated by rare high-impact tail events, so a deterministic treatment that ignores the dispersion of outcomes would systematically misjudge the required level of preparedness. These two observations, the funds–resources coupling and the heavy-tailed multi-type uncertainty, are the structural features that the next section formalizes.

### Multi-type scenario construction and risk-averse coupling of funds and resources

Building on the general description, this section introduces the minimal set of symbols needed to make clear where the contributions of this paper change the structure of the problem. The intention remains to characterize the problem rather than to develop the full model; the symbols identify the objects on which the later formulation acts. The first contribution concerns the representation of uncertainty itself. Let Ω denote the set of blackout scenarios and let ω∈Ω index an individual scenario occurring with probability $p_{\omega }$. Each scenario is not a single number but a structured outcome: it specifies a cause type k∈𝒦, with 𝒦={disaster,failure,attack}, the set of components or nodes that are damaged, the resulting load loss at each affected node, and the repair effort that restoration would require. Because a wide-area blackout can arise from many combinations of cause, location, severity and timing, a faithful description requires a very large number of such scenarios, and the three cause types must be represented jointly rather than one at a time.

The difficulty is that the cardinality of a scenario set rich enough to capture multi-type wide-area blackouts is far too high for direct optimization. The first contribution therefore frames the construction of the scenario set as a two-step problem: a massive raw set Ω is first generated, for example by Monte Carlo sampling over cause occurrence and severity, and is then condensed by scenario reduction into a representative subset $\Omega_{S}\subset \Omega$ with a recalibrated probability $\pi_{s}$ assigned to each retained scenario $s\in \Omega_{S}$, following problem-driven reduction principles for power-system stochastic optimization [40]. The reduction is governed by the requirement that the probability law carried by $\Omega_{S}$ remain close to that of Ω, while $\left|\Omega_{S}\right|$ stays small enough to be computationally tractable. This $\Omega \to \Omega_{S}$ mapping, and the trade-off between distributional fidelity and tractability that it embodies, is the structural change brought by the first contribution; the concrete reduction and sampling mechanism is developed with the model solution in the next section.

The second contribution changes the structure of the decision itself by making the financial reserve an endogenous variable coupled with the physical resources. At the first stage, the utility chooses the reserve fund scale *B* together with the pre-positioned quantity $x_{r}$ of each resource class r∈ℛ, where ℛ contains repair crews, emergency materials and spare equipment; at the second stage it chooses the scenario-dependent recourse actions, summarized for now by $y_{s}$, which deploy and supplement these resources once scenario *s* is observed. The coupling that previous studies leave implicit is made explicit by a relation of the form $\sum_{r\in ℛ}c_{r}^{pre}\;x_{r}\leq B$, which states that the cost of all pre-positioned resources is bounded by the reserve fund. This single relation captures the essence of the funds–resources interdependence: the fund *B* constrains the resources $x_{r}$ that can be staged, the resources in turn determine the recourse $y_{s}$ and hence the loss in each scenario, and the appropriate value of *B* is therefore an outcome of the downstream response it must support rather than an exogenous budget handed to the resource problem.

Not every condensation of Ω is acceptable for this problem. A reduction that matches only the mean of the loss distribution would be insufficient, because the reserve fund exists precisely to absorb the upper tail of that distribution; a representative set that smooths away rare high-severity scenarios would understate the required preparedness and defeat the purpose of the model. The reduction is therefore required to keep the retained set $\Omega_{S}$ close to Ω in a distributional sense that respects the tail, so that the worst scenarios, together with their recalibrated probabilities $\pi_{s}$, survive condensation and continue to drive the risk-averse decision. This requirement is not automatically satisfied by minimizing a generic probability-weighted distance, since a transportation-type criterion controls an average discrepancy and can trade accuracy in the sparse upper tail for accuracy in the dense body of the distribution. The Mathematical formulation section therefore states the metric, the normalization of heterogeneous attributes and the relative weighting of cause type, spatial footprint and severity explicitly, and the Case studies section reports the resulting VaR, CVaR and extreme-quantile errors against alternative reduction schemes, so that tail preservation is verified rather than assumed [34]. This requirement connects the first contribution directly with the second: the value of an accurate scenario reduction is realized only because the objective is sensitive to tail outcomes, and conversely the tail-risk objective is meaningful only if the scenario set represents the extremes faithfully.

A further structural feature brought by the second contribution is risk aversion. Minimizing only the expected second-stage loss would leave the utility exposed to the rare but severe blackout scenarios that dominate the tail of the loss distribution, which is exactly the regime that motivates holding a reserve in the first place. To control this exposure, the problem is posed so as to limit not only the average loss but also a tail-risk measure of the second-stage loss. We adopt the conditional value-at-risk at confidence level α, written ${CVaR}_{\alpha }$, which measures the expected loss in the worst (1−α) fraction of scenarios; embedding it alongside the expected cost expresses the utility’s dual concern with typical and extreme outcomes. The choice of this particular measure is deliberate. Unlike the value-at-risk, which reports only the threshold of the tail and is insensitive to how severe losses become once that threshold is crossed, the conditional value-at-risk averages over the entire tail and therefore responds to the magnitude of catastrophic blackouts, not merely their frequency. It is also a coherent risk measure with a convex, indeed linear, representation, so incorporating it preserves the computational structure of the model instead of introducing non-convexities that would compound the difficulty already caused by the integer pre-positioning decisions, which is consistent with its established use in risk-averse energy decision-making [41]. For a utility whose very motivation for holding a reserve is the possibility of rare but devastating events, a tail-averaging and tractable measure is the natural modelling choice. Locating ${CVaR}_{\alpha }$ at the problem level here makes clear that the reserve fund and the resource configuration should be chosen with explicit attention to tail losses, and prepares the ground for its formal incorporation into the objective and constraints of the model.

The information structure behind these symbols distinguishes a genuine two-stage formulation from a sequence of deterministic plans. At the moment when the reserve fund scale *B* and the pre-positioning $x_{r}$ are chosen, the utility knows only the probabilistic description of the scenarios and not which one will occur; these decisions must hedge against the whole set $\Omega_{S}$ at once. Only after a scenario *s* is realized does its damage pattern become known, and at that point the recourse $y_{s}$ is selected with full knowledge of that particular outcome. A decision rule that tacitly assumed advance knowledge of the scenario would solve a much easier and fundamentally different problem and would systematically understate the reserve and resources a utility actually needs. Preserving this non-anticipative structure, with first-stage commitments common to all scenarios and second-stage actions tailored to each, is what makes the coordinated sizing of funds and resources meaningful under uncertainty.

**Fig 2:**
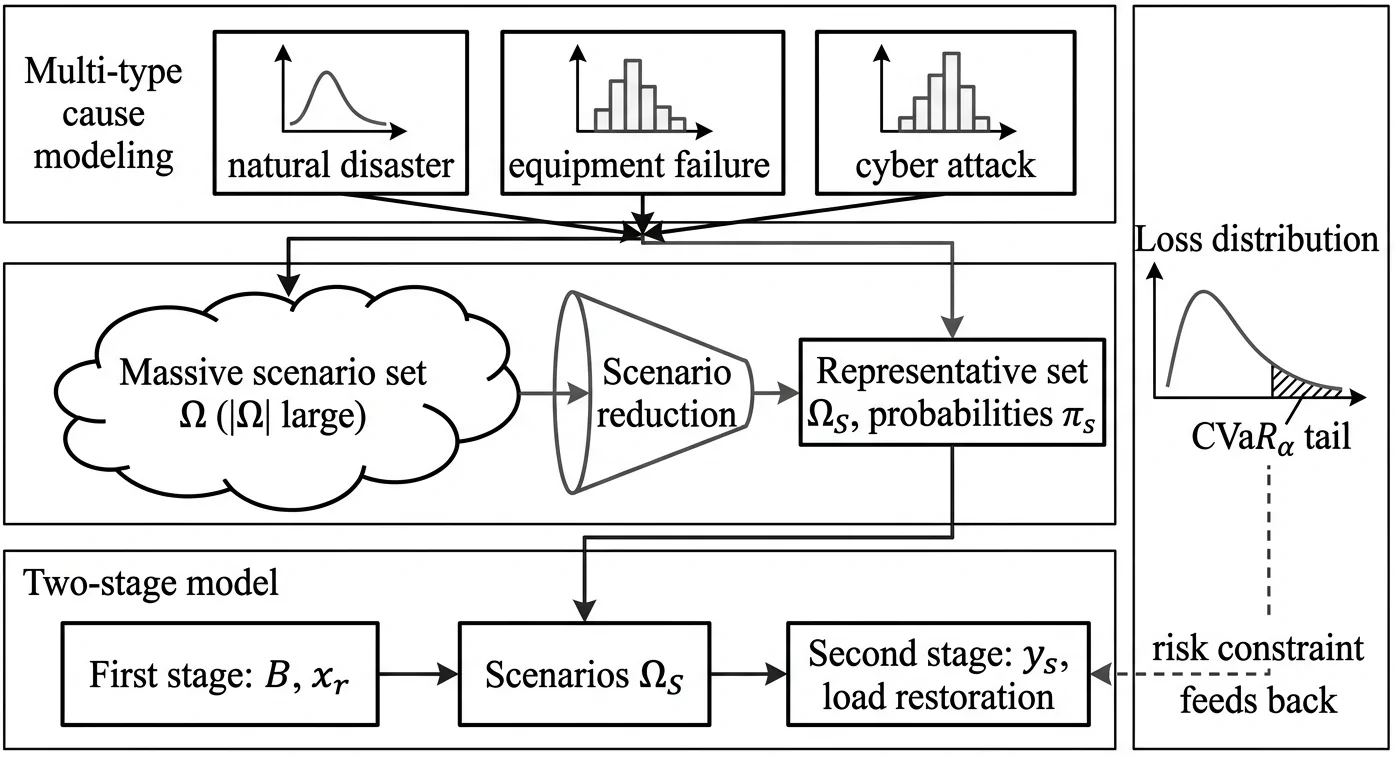
Construction and reduction of the multi-type blackout scenario set. Coupling of the retained scenario set with the two-stage risk-averse decision through the CVaR tail of the loss distribution.

Taken together, these features fix the mathematical character of the problem and its computational challenge. The decision is two-stage, with first-stage commitments that include integer-valued equipment pre-positioning, a recourse that depends on a large family of multi-type scenarios, and an objective coupled with a tail-risk measure. The resulting problem is a large-scale, risk-averse, two-stage stochastic program whose scenario count alone makes direct solution impractical, which is why the next section both establishes the model and develops a sample-average-approximation scheme with decomposition to solve it. The interplay between scenario construction and reduction on the one hand, and the risk-averse coupling of funds and resources on the other, is illustrated in Fig 2.

## Mathematical formulation

### Model establishment

This section casts the two-stage decision problem set out in the Problem description section into a risk-averse stochastic mixed-integer program. The development proceeds from the index structure to the first-stage funding and pre-positioning decisions, the time-resolved second-stage response with its resource-type-specific technology, the construction and weighting of the multi-type scenarios, and finally the risk-averse objective. Although this is the most mathematical part of the paper, each block of equations is introduced by the modelling reason that connects it to the engineering reality of blackout preparedness. Symbols are collected in Table 1.

**Table 1:** Nomenclature.

Symbol	Meaning (unit)
*Sets and indices*	
𝒩, *i*,*j*	service nodes / depot sites
𝒯, t,τ	restoration periods, |𝒯|=T, length Δt (h)
𝒦, *k*	cause types {disaster, failure, attack}
Ω, ω	raw scenario set and a raw scenario
$\Omega_{S}$, *s*	reduced (representative) scenario set and a retained scenario
$\Omega_{V}$	independent validation scenario set, $|\Omega_{V}|=N^{\prime }$
ℛ, *r*	resource classes; ${ℛ}^{ru}$ reusable, ${ℛ}^{cs}$ consumable, ${ℛ}^{cr}$ crews
*First-stage decision variables*	
*B*	reserve fund scale, i.e., pre-event preparedness capital ($M)
$x_{r,i}$	quantity of resource *r* pre-positioned at node *i* (units)
*Second-stage decision variables*	
$y_{r,i,s,t}$	quantity of resource *r* deployed at node *i* in period *t* (units)
$q_{r,s,t}$	emergency procurement of resource *r* in period *t* (units)
$z_{i,s,t}^{rp}$, $z_{i,s,t}^{en}$	load restored by repair / by re-energization (MW)
$z_{i,s,t}$	total incremental restored load at node *i* (MW)
$u_{i,s,t}$	residual unserved load at node *i* (MW)
η, $\xi_{s}^{+}$	value-at-risk threshold ($M) and scenario loss excess ($M)
$\Theta_{s}$	master-problem estimate of the scenario recourse-and-risk cost ($M)
*Parameters*	
ρ	holding (opportunity-cost) rate of the reserve (per horizon)
$B^{max}$	maximum committable capital ($M)
$c_{r,i}^{pre}$, $d_{r}^{dis}$, $e_{r}^{buy}$	unit pre-positioning / dispatch / emergency procurement cost ($M per unit)
$\kappa_{r,k}$	cause-dependent restoration productivity of resource *r* (MW per unit)
$\phi_{k}$	materials required per unit of crew effort under cause *k* (units per unit)
$\chi_{k}$	share of non-critical load that mobile equipment can re-energize
$a_{k,t}$	cause- and period-dependent reachability factor
$\ell_{r,t}$	working-hour budget per staged crew unit (units per period)
$L_{r}^{lead}$	delivery lead time of emergency procurement (periods)
$v_{i,t}^{lol}$	value of lost load at node *i* in period *t* ($/MWh)
$D_{i,s}$	realized load loss at node *i* in scenario *s* (MW)
${\overset{―}{X}}_{r,i}$, $X_{r}^{own}$	depot capacity and owned inventory of resource *r* (units)
${\overset{―}{Q}}_{r,s,t}$	emergency procurement ceiling (units)
$\pi_{s}$, $p_{\omega }$	probability of retained scenario *s* / raw scenario ω
α, β, Γ	CVaR confidence level, risk-aversion weight, CVaR ceiling ($M)
*N*, $N^{red}$, *M*	raw sample size, retained-set size, number of replications

The model is defined on five index sets. Nodes or service zones form the set 𝒩, indexed by *i* and *j*; the response horizon is discretized into periods 𝒯={1,...,T}, indexed by *t*; cause types form 𝒦, indexed by *k*; and the reduced scenario set is $\Omega_{S}$, indexed by *s* with probabilities $\pi_{s}$. Emergency resources form the set ℛ, par*t*itioned into reusable classes ${ℛ}^{ru}$, comprising repair crews and spare equipment, and consumable classes ${ℛ}^{cs}$, comprising materials such as poles, conductors and fuel that are used up as restoration proceeds; crews form the subset ${ℛ}^{cr}\subseteq {ℛ}^{ru}$ and mobile equipment the subset ${ℛ}^{eq}\subseteq {ℛ}^{ru}$, with ${ℛ}^{cr}\cap {ℛ}^{eq}=\emptyset$. The first-stage decisions are the reserve fund scale B≥0 and the pre-positioned quantity $x_{r,i}\geq 0$ of resource *r* staged at node *i*, whose spare-equipment components are integer-valued. The recourse decisions, taken after scenario *s* is observed and resolved period by period, are the deployed quantity $y_{r,i,s,t}$, the emergency procurement $q_{r,i,s,t}$ delivered to node *i*, the incremental restored load $z_{i,s,t}$, and the residual unserved load $u_{i,s,t}$. Each scenario carries a unique cause label, written *k*(*s*), on which the restoration technology depends.

The first stage fixes the preparedness posture before any event is known. The total preparation cost committed by the utility is the opportunity cost of holding the reserve together with the cost of staging every resource class over all nodes, and this constitutes the deterministic part of the objective,


$$\begin{array}{c} C^{pre}\;=\;\rho \;B\;+\;\sum_{r\in ℛ}\sum_{i\in 𝒩}c_{r,i}^{pre}\;x_{r,i}. \end{array}$$ (1)

The accounting interpretation of the two terms in (1) requires care, because they refer to different economic quantities and must not be confused. The variable *B* denotes the total pre-event preparedness capital that the utility commits to the emergency function at the start of the horizon. Of that commitment, the amount $\sum_{r,i}c_{r,i}^{pre}x_{r,i}$ is converted into physical assets before any event occurs and is therefore an acquisition expenditure, while the remainder $B-\sum_{r,i}c_{r,i}^{pre}x_{r,i}$ is retained as liquid capital and remains available to finance post-event procurement. The term ρB is the opportunity cost of committing the whole amount *B* to the emergency function rather than to an alternative use over the preparedness horizon, and it is charged on the full commitment because both the converted and the retained portions are withdrawn from alternative use. No quantity is counted twice: ρB prices the commitment of capital and $\sum_{r,i}c_{r,i}^{pre}x_{r,i}$ prices the assets purchased with part of it, and the unconverted residual is not itself an expense and does not enter (1). Dispatch cost is treated as an operating expenditure charged against the second stage in (21) rather than against the reserve, on the ground that the reserve is held to finance capital acquisition and emergency purchases in a strained post-event market, whereas crew hours and fuel consumed during a response are recovered through ordinary operating budgets; this asymmetry is a modelling convention and is stated here so that it can be varied by a user who accounts differently.

The staging of resources must be financed within a reserve that is itself capped by the utility’s maximum committable capital, and this is the financial backbone of the here-and-now decision,


$$\begin{array}{c} \sum_{r\in ℛ}\sum_{i\in 𝒩}c_{r,i}^{pre}\;x_{r,i}\;\leq \;B\;\leq \;B^{max}. \end{array}$$ (2)

Pre-positioning is further limited by depot capacity at each node and by the utility’s owned inventory of each resource class, so that staging respects both local and system-wide physical bounds,


$$\begin{array}{c} x_{r,i}\;\leq \;{\overset{―}{X}}_{r,i}\;\;\forall r\in ℛ,\;i\in 𝒩;\;\sum_{i\in 𝒩}x_{r,i}\;\leq \;X_{r}^{own}\;\;\forall r\in ℛ, \end{array}$$ (3)

where the spare-equipment components of $x_{r,i}$ satisfy $x_{r,i}\in {\mathbb{Z}}_{+}$ and all other components are continuous and non-negative.

Once scenario *s* occurs, the utility restores load over the horizon 𝒯 within the envelope set by its first-stage choices, and the distinction between reusable and consumable resources is essential for physical realism, in agreement with coordinated post-event restoration models for transmission and distribution systems [42]. A repair crew or a mobile generator deployed in one period is released for redeployment in the next, so the quantity of a reusable resource dispatched in any single period cannot exceed the staged stock plus whatever has been procured and delivered by that period,


$$\begin{array}{c} \sum_{i\in 𝒩}y_{r,i,s,t}\;\leq \;\sum_{i\in 𝒩}x_{r,i}\;+\;\sum_{\tau \leq t-L_{r}^{lead}}\sum_{i\in 𝒩}q_{r,i,s,\tau },\;\forall r\in {ℛ}^{ru},\;s\in \Omega_{S},\;t\in 𝒯. \end{array}$$ (4)

A consumable resource, by contrast, is used up when deployed, so it is the cumulative deployment over the horizon that the staged stock and the delivered procurement must cover,


$$\begin{array}{c} \sum_{\tau \leq t}\sum_{i\in 𝒩}y_{r,i,s,\tau }\;\leq \;\sum_{i\in 𝒩}x_{r,i}\;+\;\sum_{\tau \leq t-L_{r}^{lead}}\sum_{i\in 𝒩}q_{r,i,s,\tau },\;\forall r\in {ℛ}^{cs},\;s\in \Omega_{S},\;t\in 𝒯. \end{array}$$ (5)

The technology that converts deployed resources into restored load is the point at which the three resource classes cease to be interchangeable, and it is specified here as two distinct channels rather than as a single additive capability term. The first channel is physical repair, which requires crews and materials jointly: crew effort without the corresponding hardware restores nothing, and stocked hardware without crews to install it restores nothing either. Repair output at a node is therefore bounded separately by the crew effort deployed there and by the materials consumed there, so that the binding input is whichever of the two is scarcer,


$$\begin{array}{c} z_{i,s,t}^{rp}\;\leq \;\sum_{r\in {ℛ}^{cr}}\kappa_{r,k(s)}\;a_{k(s),t}\;y_{r,i,s,t},\;\forall i\in 𝒩,\;s\in \Omega_{S},\;t\in 𝒯, \end{array}$$ (6)

$$\begin{array}{c} z_{i,s,t}^{rp}\;\leq \;\frac{1}{\phi_{k(s)}}\sum_{r\in {ℛ}^{cs}}\kappa_{r,k(s)}\;y_{r,i,s,t},\;\forall i\in 𝒩,\;s\in \Omega_{S},\;t\in 𝒯. \end{array}$$ (7)

Constraints (6) and (7) together implement a Leontief-type repair technology, since at an optimal solution the restored quantity equals the minimum of the two right-hand sides and each input is therefore necessary and non-substitutable; the parameter $\phi_{k}$ fixes the material requirement per unit of crew effort under cause *k*, and raising the crew count without a proportionate material stock leaves $z^{rp}$ unchanged. This is the property that the additive form used in the earlier formulation could not reproduce.

The second channel is temporary re-energization by mobile equipment, which bypasses physical repair rather than accelerating it. Mobile generators and transformers restore supply directly, consume no repair materials, and are the instrument on which cyber-induced outages depend, since those outages disable control and communication functions without necessarily destroying plant. Re-energization output is limited by the equipment deployed and by its cause-dependent effectiveness,


$$\begin{array}{c} z_{i,s,t}^{en}\;\leq \;\sum_{r\in {ℛ}^{eq}}\kappa_{r,k(s)}\;a_{k(s),t}^{en}\;y_{r,i,s,t},\;\forall i\in 𝒩,\;s\in \Omega_{S},\;t\in 𝒯. \end{array}$$ (8)

Mobile equipment cannot serve an arbitrary share of the affected load, because back-feeding a node requires switching capability and local capacity that are available for critical load in every regime but only partially available for ordinary load, and to a degree that depends on the cause. Writing $D_{i,s}^{cr}$ and $D_{i,s}^{nc}$ for the critical and non-critical components of the nodal loss, the cumulative re-energized quantity is capped by


$$\begin{array}{c} \sum_{t\in 𝒯}z_{i,s,t}^{en}\;\leq \;D_{i,s}^{cr}\;+\;\chi_{k(s)}\;D_{i,s}^{nc},\;\forall i\in 𝒩,\;s\in \Omega_{S}. \end{array}$$ (9)

The coverage share $\chi_{k}$ is small under natural disasters, where the plant itself is destroyed and physical repair is unavoidable, intermediate under equipment failures, and large under cyber attacks, where the primary asset is intact and back-feeding restores supply. Together with the cause-indexed productivities $\kappa_{r,k}$ and reachability factors $a_{k,t}$ and $a_{k,t}^{en}$, this is what makes the restoration technology genuinely cause-specific: crews are highly effective against equipment failures, are impeded early in a disaster and ramp up as access is restored, and contribute little against a cyber-induced outage, while equipment is decisive in the cyber regime. The total incremental restoration at a node is the sum of the two channels,


$$\begin{array}{c} z_{i,s,t}\;=\;z_{i,s,t}^{rp}\;+\;z_{i,s,t}^{en},\;\forall i\in 𝒩,\;s\in \Omega_{S},\;t\in 𝒯. \end{array}$$ (10)

Crews additionally face a labour-capacity ceiling, because a finite workforce can mobilize only a bounded effort per period however much is nominally on hand; with $\ell_{r,t}$ the working-hour budget per staged crew unit, the per-period crew effort is limited by


$$\begin{array}{c} \sum_{i\in 𝒩}y_{r,i,s,t}\;\leq \;\ell_{r,t}\sum_{i\in 𝒩}x_{r,i},\;\forall r\in {ℛ}^{cr},\;s\in \Omega_{S},\;t\in 𝒯. \end{array}$$ (11)

Restoration is cumulative and cannot exceed the load actually lost at the node, which couples the recourse to the scenario data and enforces monotone clearing of the outage,


$$\begin{array}{c} 0\;\leq \;\sum_{\tau \leq t}z_{i,s,\tau }\;\leq \;D_{i,s},\;\forall i\in 𝒩,\;s\in \Omega_{S},\;t\in 𝒯. \end{array}$$ (12)

The unserved load in each period is the part of the realized loss not yet recovered by that period, and it is this quantity that carries the value-of-lost-load penalty in the objective,


$$\begin{array}{c} u_{i,s,t}\;=\;D_{i,s}\;-\;\sum_{\tau \leq t}z_{i,s,\tau },\;\forall i\in 𝒩,\;s\in \Omega_{S},\;t\in 𝒯. \end{array}$$ (13)

Restoration is useful only where resources can reach an affected node within the access conditions and the critical time window of the scenario, so deployment to a node is restricted to origins able to serve it, expressed by the binary reachability field $a_{j,i,s,t}$, together with procurement already delivered to that node,


$$\begin{array}{c} y_{r,i,s,t}\;\leq \;\sum_{j\in 𝒩}a_{j,i,s,t}\;x_{r,j}\;+\;\sum_{\tau \leq t-L_{r}^{lead}}q_{r,i,s,\tau },\;\forall r\in ℛ,\;i\in 𝒩,\;s\in \Omega_{S},\;t\in 𝒯. \end{array}$$ (14)

Indexing procurement by its destination node removes an ambiguity of the earlier formulation, in which a single system-level procurement quantity appeared on the right-hand side of the reachability constraint of every node and could therefore support deployment at several destinations at once. With $q_{r,i,s,t}$ attached to a destination, the same procured unit relaxes the reachability constraint of exactly one node, and the aggregate limit imposed by a strained post-event market applies to the sum over destinations,


$$\begin{array}{c} q_{r,i,s,t}\;\geq \;0;\;\sum_{i\in 𝒩}\sum_{t\in 𝒯}q_{r,i,s,t}\;\leq \;{\overset{―}{Q}}_{r,s},\;\forall r\in ℛ,\;s\in \Omega_{S}. \end{array}$$ (15)

Importantly for the funds–resources coupling, the cost of procurement in every scenario is financed from whatever portion of the reserve is not already committed to pre-positioning, which binds the second-stage actions back to the first-stage reserve decision,


$$\begin{array}{c} \sum_{t\in 𝒯}\sum_{i\in 𝒩}\sum_{r\in ℛ}e_{r}^{buy}\;q_{r,i,s,t}\;\leq \;B\;-\;\sum_{r\in ℛ}\sum_{i\in 𝒩}c_{r,i}^{pre}\;x_{r,i},\;\forall s\in \Omega_{S}. \end{array}$$ (16)

Imposing (16) for every retained scenario has a definite economic meaning that should be stated rather than left implicit. Since the scenarios are mutually exclusive realizations and the residual reserve is not consumed until one of them occurs, the same liquid balance is indeed available whichever scenario materializes, so the constraint does not double-spend the reserve across scenarios. It does, however, require the residual balance to cover the largest scenario-specific procurement requirement in the retained set, irrespective of that scenario’s probability, and it therefore behaves as a robust liquidity requirement layered on top of the CVaR objective. The consequence is that a single rare but procurement-intensive retained scenario can raise *B* on its own. A less conservative alternative is a chance-constrained requirement, in which (16) is imposed with probability at least 1−ε, or a risk-weighted requirement in which the expected or tail-average procurement outlay rather than its maximum must be financed. Both alternatives lower the reserve and shift part of the liquidity risk onto the utility; the robust form is retained here because it corresponds to the practice of guaranteeing that committed emergency financing is never exhausted mid-response, and because it makes the funds–resources coupling binding rather than slack in the majority of scenarios.

The multi-type character of the uncertainty enters through the way scenario data are generated and weighted. Each retained scenario is assigned to exactly one cause type by the indicator $\theta_{k,s}\in \{0,1\}$, and the load loss at a node is then drawn from the conditional model of that cause through the cause-specific exposure $g_{i,k}$ and the severity $\delta_{k,s}$, so that the spatial footprint and magnitude of the outage reflect whether it originates in a natural disaster, an equipment failure or a cyber attack,


$$\begin{array}{c} \sum_{k\in 𝒦}\theta_{k,s}\;=\;1\;\;\forall s\in \Omega_{S};\;D_{i,s}\;=\;\sum_{k\in 𝒦}\theta_{k,s}\;g_{i,k}\;\delta_{k,s},\;\forall i\in 𝒩,\;s\in \Omega_{S}. \end{array}$$ (17)

The assignment in (17) is exclusive, so the scenario set is a mixture of single-cause events and not a model of compound or interacting hazards. This restriction is made explicit here because the terminology of multi-type risk could otherwise be read as covering joint occurrences. Natural, technical and cyber disruptions are in reality dependent: a storm can trigger an equipment cascade, and an intrusion is more damaging when it coincides with a weather event that has already consumed field resources. Under exclusivity the model understates the upper tail, since the most severe compound realizations are absent from the scenario set by construction. The Case studies section quantifies the resulting error by evaluating both the single-cause plan and a compound-aware plan on validation sets in which a second cause is superimposed with probability $p^{cp}$, so that the cost of the assumption is measured rather than asserted. Extending (17) to a joint cause model, for example through a copula on cause occurrence and severity, is a direct generalization that leaves the remaining formulation unchanged.

Because the retained set $\Omega_{S}$ is condensed from the massive raw set Ω, its probabilities must form a valid law inheriting the mass of the raw scenarios assigned to each representative during reduction, through the partition $\{\Omega^{\left(s\right)}{\}}_{s\in \Omega_{S}}$ of Ω,


$$\begin{array}{c} \pi_{s}\;=\;\sum_{\omega \in \Omega^{\left(s\right)}}p_{\omega }\;\geq \;0\;\;\forall s\in \Omega_{S},\;\sum_{s\in \Omega_{S}}\pi_{s}\;=\;1. \end{array}$$ (18)

The reduction itself is not arbitrary: it selects the representative set and the assignment so as to minimize the probability-weighted distance between each raw scenario and the representative to which it is mapped, a transportation-type criterion that keeps $\Omega_{S}$ close to Ω in distribution,


$$\begin{array}{c} \min_{\Omega_{S}\subset \Omega ,\;\{\Omega^{\left(s\right)}\}}\;\;\sum_{s\in \Omega_{S}}\sum_{\omega \in \Omega^{\left(s\right)}}p_{\omega }\;\backslash lVert\xi_{\omega }-\xi_{s}{\backslash rVert}_{2}\;\text{s.t.}\;\;|\Omega_{S}|\leq N^{red}. \end{array}$$ (19)

The metric in (19) is stated explicitly, because a transportation criterion is only as tail-aware as the attribute space and normalization on which it is computed. The attribute vector of a scenario combines four continuous damage descriptors with a categorical cause label,


$$\begin{array}{c} \xi_{\omega }\;=\;(\frac{\Lambda_{\omega }}{\sigma_{\Lambda }}\lambda_{1},\;\frac{n_{\omega }}{\sigma_{n}},\;\frac{P_{\omega }}{\sigma_{P}}\lambda_{2},\;\frac{\varsigma_{\omega }}{\sigma_{\varsigma }},\;\frac{1[k(\omega )=1]}{\sigma_{1}},\;\frac{1[k(\omega )=2]}{\sigma_{2}},\;\frac{1[k(\omega )=3]}{\sigma_{3}}), \end{array}$$ (20)

where $\Lambda_{\omega }=\sum_{i}D_{i,\omega }$ is the total load loss, $n_{\omega }$ the number of affected nodes, $P_{\omega }=\max_{i}D_{i,\omega }$ the peak nodal loss, $\varsigma_{\omega }$ the loss-weighted spatial spread of the footprint, and the last three entries one-hot encode the cause. Each component is divided by its empirical standard deviation over Ω, so that heterogeneous attributes enter on a common scale and the Euclidean norm in (19) is well posed; the one-hot components acquire a comparatively large weight through this normalization, since a rare cause has a small standard deviation, which is the mechanism by which cause type is prevented from being averaged away. The two factors $\lambda_{1}=1.6$ and $\lambda_{2}=1.3$ deliberately over-weight total and peak loss relative to footprint and cause, which is what makes the criterion tail-aware: severity differences in the sparse upper region of the distribution are magnified, so severe scenarios are less readily absorbed into a cluster dominated by mild ones and tend to be retained as their own representatives. Problem (19) is NP-hard, and it is solved here by *k*-means++ initialization followed by Lloyd iterations in the space defined by (20), after which the medoid of each cluster, that is the raw scenario closest to the cluster centre, is retained as the representative so that every $\xi_{s}$ is an attainable outcome rather than a synthetic average, and $\pi_{s}$ is set by (18). Because this construction guarantees tail fidelity by design rather than by proof, the Case studies section measures it directly, reporting VaR, CVaR and extreme-quantile errors against the raw empirical distribution, together with the out-of-sample regret of the resulting first-stage decision, and comparing them with random subsampling, medoid clustering without the tail weights, and fast-forward selection [3 4].

With the recourse fully described, the loss in a scenario aggregates over the horizon the cost of dispatching resources, the cost of emergency procurement, and the time-weighted value of load left unserved, where the weight $v_{i,t}^{lol}$ rises with outage duration so as to reflect the growing harm of a prolonged interruption,


$$\begin{array}{c} L_{s}\;=\;\sum_{t\in 𝒯}[\sum_{r\in ℛ}\sum_{i\in 𝒩}d_{r}^{dis}\;y_{r,i,s,t}\;+\;\sum_{r\in ℛ}\sum_{i\in 𝒩}e_{r}^{buy}\;q_{r,i,s,t}\;+\;\sum_{i\in 𝒩}v_{i,t}^{lol}\;u_{i,s,t}\;\Delta t],\;\forall s\in \Omega_{S}. \end{array}$$ (21)

The utility’s overall expected cost combines the first-stage preparation cost with the probability-weighted second-stage loss; for a risk-neutral utility this expectation would form the whole objective,


$$\begin{array}{c} C^{exp}\;=\;C^{pre}\;+\;\sum_{s\in \Omega_{S}}\pi_{s}\;L_{s}. \end{array}$$ (22)

The model must penalize the tail of the loss distribution and not only its mean. Adopting the conditional value-at-risk at confidence level α in its Rockafellar–Uryasev form, the value-at-risk threshold η and the non-negative excess $\xi_{s}^{+}$ measuring the exceedance of the loss over that threshold are linked by


$$\begin{array}{c} \xi_{s}^{+}\;\geq \;L_{s}-\eta ,\;\xi_{s}^{+}\;\geq \;0,\;\forall s\in \Omega_{S}, \end{array}$$ (23)

and the conditional value-at-risk is then the threshold plus the scaled expected exceedance over the retained scenarios,


$$\begin{array}{c} {CVaR}_{\alpha }\;=\;\eta \;+\;\frac{1}{1-\alpha }\sum_{s\in \Omega_{S}}\pi_{s}\;\xi_{s}^{+}. \end{array}$$ (24)

The risk-averse objective trades off expected cost against this tail term through a risk-aversion weight β≥0, where β=0 recovers the risk-neutral case; the utility minimizes the resulting risk-adjusted total cost over all first- and second-stage variables,


$$\begin{array}{c} \min_{B,\;x_{r,i},\;y_{r,i,s,t},\;q_{r,i,s,t},\;z_{i,s,t},\;u_{i,s,t},\;\eta ,\;\xi_{s}^{+}}\;\;C^{pre}\;+\;\sum_{s\in \Omega_{S}}\pi_{s}\;L_{s}\;+\;\beta \left(\eta +\frac{1}{1-\alpha }\sum_{s\in \Omega_{S}}\pi_{s}\;\xi_{s}^{+}\right). \end{array}$$ (25)

A hard ceiling Γ on the conditional value-at-risk may additionally be imposed to express a regulatory or board-mandated tolerance for extreme losses, which lets the risk attitude be enforced rather than merely priced through β,


$$\begin{array}{c} \eta \;+\;\frac{1}{1-\alpha }\sum_{s\in \Omega_{S}}\pi_{s}\;\xi_{s}^{+}\;\leq \;\Gamma . \end{array}$$ (26)

Finally, a reliability indicator summarizing the preparedness outcome, reported in the case studies, is the expected unserved energy accumulated over nodes, periods and scenarios, with Δt the period length,


$$\begin{array}{c} EUE\;=\;\sum_{s\in \Omega_{S}}\pi_{s}\sum_{t\in 𝒯}\sum_{i\in 𝒩}u_{i,s,t}\;\Delta t. \end{array}$$ (27)

Equations (1)–(27), together with the non-negativity of all continuous variables and the integrality of the spare-equipment components of $x_{r,i}$, constitute a two-stage risk-averse stochastic mixed-integer linear program. The first-stage variables *B* and $x_{r,i}$ are common to all scenarios, while the time-indexed recourse and the risk variables adapt to each scenario; the objective and the residual-financing constraint (16) bind the two stages together, and the size of the program grows linearly with $\left|\Omega_{S}|\;|𝒯\right|$. Because every relation above is linear in the decision variables, no auxiliary linearization is needed.

Three modelling assumptions delimit what the formulation can support, and they are recorded here so that the results of the Case studies section are read within the correct scope. First, reusable resources are released at the end of each period and are available again in the next, so travel time between depot and worksite, service duration on a multi-period task, return trips and the resulting occupancy states are not represented. Delay is captured only in aggregate, through the cause- and period-dependent reachability factors $a_{k,t}$ and $a_{k,t}^{en}$, which suppress early-period productivity when access is obstructed. This treatment is optimistic relative to a model with explicit occupancy, since a crew that would in reality remain committed to one task for several periods is free to redeploy here; the consequence is that the absolute restoration speed reported below should be read as an upper envelope, while the comparisons between preparedness plans, which are the object of this study, are affected symmetrically because every plan is evaluated under the same technology. Second, restoration is represented at the level of aggregate served load per unit of resource through $\kappa_{r,k}$ rather than through a power-flow model, so network operability, voltage limits, line capacities and islanding play no part; the network supplies spatial layout and reachability only. Third, the reserve is a single liquid pool priced by the rate ρ, which abstracts from financing frictions so that the funds–resources coupling stands out.

The computational study in the Case studies section solves an aggregated form of this program, and the aggregation is stated precisely so that the reported quantities can be interpreted correctly. The nodal loss vector of each scenario is partitioned into a critical and a non-critical tier, each carrying the corresponding mean value of lost load, and the recourse is resolved on those two tiers over the 24 periods rather than on all 118 nodes individually, with restoration allocated to the critical tier first. Deployment is correspondingly represented by system-level quantities subject to the cause-dependent reachability profiles, so that constraints (6)–(11) act on the aggregate flow of each channel and (14) is enforced in its aggregate form. The network data that enter the optimization are therefore the nodal loads, the critical-node designation, the loss footprints generated per scenario and the reachability profiles; the coordinates, edges and depot sites determine the spatial structure of those footprints and are used in the scenario generator, but no line-level quantity enters the optimization. This aggregation is what makes the replication and validation scheme of the next subsection affordable, and it is consistent with the strategic scope declared in the Problem description section; a node-resolved solution of (1)–(27) is a direct but computationally heavier instance of the same formulation.

### Model solution

The model established above is a mixed-integer linear program, but it cannot be solved by enumerating all blackout outcomes. The difficulty originates in the description of uncertainty itself: a faithful representation of multi-type wide-area blackouts requires an enormous raw scenario set Ω, and both the expectation and the conditional value-at-risk in the objective (25) are defined with respect to it. Evaluating these quantities over the full Ω, and optimizing the integer first-stage decisions against it, is intractable, because the number of recourse blocks scales with the scenario count multiplied by the horizon length. The solution method must therefore reconcile the statistical need for many scenarios with the computational need for few, which is exactly the tension that the scenario construction of the Problem description section framed and that the sample average approximation resolves here.

The procedure mirrors the $\Omega \to \Omega_{S}$ mapping in two coupled steps. A finite sample of scenarios is first drawn by Monte Carlo simulation from the multi-type probability model, each draw fixing a cause type through $\theta_{k,s}$ and the associated loss pattern $D_{i,s}$ and reachability field; the raw sample is then condensed by the reduction criterion (19) into the representative set $\Omega_{S}$ with recalibrated probabilities (18). The expectation and the conditional value-at-risk are subsequently replaced by their sample averages over $\Omega_{S}$, which turns the intractable stochastic program into a finite mixed-integer program whose optimal value, for a raw sample of size *N* reduced to $N^{red}$ representatives, is


$$\begin{array}{c} {\hat{v}}_{N}^{red}\;=\;\min_{B,\;x_{r,i}\in 𝒳}\;\;C^{pre}\;+\;\sum_{s\in \Omega_{S}}\pi_{s}\;L_{s}\;+\;\beta \left(\eta +\frac{1}{1-\alpha }\sum_{s\in \Omega_{S}}\pi_{s}\;\xi_{s}^{+}\right), \end{array}$$ (28)

where 𝒳 denotes the first-stage feasible set defined by (2)–(3) and the recourse for each retained scenario is the cost of solving (4)–(17), (21) and (23).

The statistical status of (28) requires care, and the earlier version of this work did not distinguish it sharply enough. Classical sample-average-approximation theory establishes that the optimal value of a problem built on an independent and identically distributed sample is a downward-biased estimator of the true optimal value, so that the average of such optimal values over independent replications estimates a valid lower bound, and that it converges as the sample size grows [43]. That argument applies to Ω, not to $\Omega_{S}$: the reduced set is produced by a data-dependent optimization (19) and carries non-uniform weights (18), so it is not an ordinary independent sample and the lower-bound property is not inherited. A valid lower-bound estimator is therefore constructed on unreduced samples. Solving *M* independently drawn raw samples of size *N* without reduction yields


$$\begin{array}{c} {\bar{L}}_{N,M}^{raw}\;=\;\frac{1}{M}\sum_{m=1}^{M}{\hat{v}}_{N}^{\left(m\right)},\;\hat{se}({\bar{L}}_{N,M}^{raw})\;=\;[\frac{1}{M(M-1)}\sum_{m=1}^{M}({\hat{v}}_{N}^{\left(m\right)}-{\bar{L}}_{N,M}^{raw}{)}^{2}{]}^{1/2}, \end{array}$$ (29)

which is the quantity used below whenever a lower bound is invoked. The corresponding average of reduced-set optimal values, ${\bar{L}}_{N,M}^{red}$, is retained only as a diagnostic, and their difference


$$\begin{array}{c} {\hat{b}}_{N}\;=\;{\bar{L}}_{N,M}^{red}\;-\;{\bar{L}}_{N,M}^{raw} \end{array}$$ (30)

estimates the bias that reduction introduces into the in-sample objective. Reporting ${\hat{b}}_{N}$ separately is what allows the approximation error of the reduction to be distinguished from the sampling error of the Monte Carlo draw.

A candidate first-stage solution $\left(\hat{B},{\hat{x}}_{r,i}\right)$ obtained from one instance is then fixed and evaluated on a large independent validation set $\Omega_{V}$ of $N^{\prime }$ scenarios, which gives an estimator of the cost the utility would actually incur,


$$\begin{array}{c} {\hat{U}}_{N^{\prime }}\;=\;{\hat{C}}^{pre}\;+\;\frac{1}{N^{\prime }}\sum_{s\in \Omega_{V}}L_{s}(\hat{B},{\hat{x}}_{r,i})\;+\;\beta \;{\hat{CVaR}}_{\alpha }(\{L_{s}{\}}_{s\in \Omega_{V}}). \end{array}$$ (31)

The expectation term in (31) is unbiased for the true expected recourse cost of the candidate policy, since that policy is fixed before $\Omega_{V}$ is drawn, and the whole expression is an upper bound in expectation for the optimal value of the true problem. The empirical CVaR term is a plug-in quantile-based estimator and is consistent but not unbiased in finite samples, so (31) is described here as a consistent upper-bound estimator rather than an unbiased one, and its sampling error is reported alongside the point estimate. The quality of the candidate is then measured by the difference between the two estimators, reported with a confidence interval whose half-width follows from their standard errors at the normal quantile $z_{1-\gamma /2}$,


$$\begin{array}{c} {\hat{gap}}_{N}\;=\;{\hat{U}}_{N^{\prime }}-{\bar{L}}_{N,M}^{raw}\;\pm \;z_{1-\gamma /2}\sqrt{{\hat{se}}^{2}({\hat{U}}_{N^{\prime }})+{\hat{se}}^{2}({\bar{L}}_{N,M}^{raw})}. \end{array}$$ (32)

Four error sources are thereby kept apart and are quantified separately in the Case studies section. Monte Carlo sampling error is the dispersion of ${\hat{v}}_{N}^{\left(m\right)}$ across independent raw samples and is measured by the replication standard deviation underlying (29). Scenario-reduction error is the systematic component ${\hat{b}}_{N}$ of (30). Optimization error is the dispersion of the returned objective across independent starts of the first-stage search on one fixed instance, and it is not a sampling quantity at all. Out-of-sample evaluation error is the dispersion of ${\hat{U}}_{N^{\prime }}$ across independent validation sets. Only the first and the last are reflected in the interval of (32); reporting the other two alongside prevents an interval that is narrow for sampling reasons from being read as evidence that the solution is close to optimal in every respect.

The overall procedure draws an initial Monte Carlo batch and reduces it, builds and solves the sample average approximation (28) to obtain a candidate reserve and pre-positioning plan, estimates the bounds (29)–(32), and refines the sample only as far as the desired accuracy demands. Its logic is summarized as follows.

1) Input the multi-type cause models, the cost, capability and capacity parameters, the confidence level α, the risk weight β and tolerance Γ, the initial sample size *N*, the replication count *M*, the validation size $N^{\prime }$, and the gap tolerance. 2) Draw a Monte Carlo batch from the cause models and reduce it via (19) to a representative set $\Omega_{S}$ with probabilities (18). 3) Construct and solve the sample average approximation (28) over $\Omega_{S}$ to obtain $\left(\hat{B},{\hat{x}}_{r,i}\right)$ and ${\hat{v}}_{N}^{red}$. 4) Repeat steps 2–3 over *M* replications, solve the same *M* raw samples without reduction to form ${\bar{L}}_{N,M}^{raw}$ in (29) and the bias ${\hat{b}}_{N}$ in (30), and evaluate the fixed $\left(\hat{B},{\hat{x}}_{r,i}\right)$ on $\Omega_{V}$ to form ${\hat{U}}_{N^{\prime }}$ in (31). 5) If ${\hat{gap}}_{N}$ in (32) is within tolerance, return $\left(\hat{B},{\hat{x}}_{r,i}\right)$ and the risk metrics; otherwise increase *N* and return to step 2.

When the representative set required for an accurate approximation is large, the two-stage structure invites a decomposition that exploits it, and the derivation is given here in full because the schematic cut of the earlier version was not a valid Benders cut. Collect the first-stage variables in 𝐰=(B,𝐱) and the recourse variables of scenario *s*, including its risk excess, in ${𝐯}_{s}=({𝐲}_{s},{𝐪}_{s},{𝐳}_{s},{𝐮}_{s},\xi_{s}^{+})$. Constraints (4)–(17) and (23) are linear in $\left(𝐰,\eta ,{𝐯}_{s}\right)$ and separate across scenarios once (𝐰,η) is fixed, so the scenario subproblem is


$$\begin{array}{c} Q_{s}(𝐰,\eta )\;=\;\min_{{𝐯}_{s}\geq 0}\;\backslash \{\;\pi_{s}\;{𝐜}_{s}^{⊤}{𝐯}_{s}\;:\;{𝐖}_{s}{𝐯}_{s}\;\geq \;{𝐡}_{s}+{𝐓}_{s}𝐰+{𝐭}_{s}\;\eta \;\backslash \}, \end{array}$$ (33)

where ${𝐜}_{s}$ collects the dispatch, procurement and value-of-lost-load coefficients of (21) together with the coefficient $\beta \pi_{s}/(1-\alpha )$ attached to $\xi_{s}^{+}$, the rows of ${𝐓}_{s}$ carry the staged quantities into the availability and reachability constraints (4), (5), (11) and (14) and the residual balance into (16), and ${𝐭}_{s}$ carries the threshold η into the risk row (23). Placing $\xi_{s}^{+}$ inside the subproblem, rather than in the master, is what keeps the CVaR term decomposable: the master retains only the scalar η, and the tail contribution of each scenario is priced through its own subproblem. The recourse is complete, since setting ${𝐲}_{s}={𝐪}_{s}={𝐳}_{s}=0$, ${𝐮}_{s}={𝐃}_{s}$ and $\xi_{s}^{+}=\max\{L_{s}-\eta ,0\}$ is feasible for any (𝐰,η) satisfying (2)–(3); consequently $Q_{s}$ is finite everywhere on the master feasible set and no feasibility cut is ever required. By linear programming duality,


$$\begin{array}{c} Q_{s}(𝐰,\eta )\;=\;\max_{\lambda_{s}\geq 0}\;\backslash \{\;\lambda_{s}^{⊤}({𝐡}_{s}+{𝐓}_{s}𝐰+{𝐭}_{s}\eta )\;:\;{𝐖}_{s}^{⊤}\lambda_{s}\;\leq \;\pi_{s}{𝐜}_{s}\;\backslash \}, \end{array}$$ (34)

so $Q_{s}$ is a pointwise maximum of affine functions of (𝐰,η) and is therefore convex and piecewise linear in the master variables. Let ${\hat{\lambda }}_{s}$ be an optimal dual solution of (33) at the incumbent $\left(\hat{𝐰},\hat{\eta }\right)$. Weak duality applied to (34) gives $Q_{s}(𝐰,\eta )\geq {\hat{\lambda }}_{s}^{⊤}({𝐡}_{s}+{𝐓}_{s}𝐰+{𝐭}_{s}\eta )$ for every (𝐰,η), which yields the optimality cut


$$\begin{array}{c} \Theta_{s}\;\geq \;{\hat{\lambda }}_{s}^{⊤}{𝐡}_{s}\;+\;({𝐓}_{s}^{⊤}{\hat{\lambda }}_{s}{)}^{⊤}𝐰\;+\;({𝐭}_{s}^{⊤}{\hat{\lambda }}_{s})\;\eta ,\;\forall s\in \Omega_{S}. \end{array}$$ (35)

Unlike the schematic expression it replaces,(35) is affine in the current master variables 𝐰 and η, contains no already-evaluated recourse value and no separately added risk term, and therefore constrains future master solutions rather than merely restating the incumbent cost. One cut per scenario is generated at each iteration, which is the multi-cut variant; it produces a tighter master relaxation than a single aggregated cut at the price of $\left|\Omega_{S}\right|$ additional rows per iteration, and this trade favours the multi-cut form here because the subproblems are small and numerous. The master problem accumulates these cuts,


$$\begin{array}{cc} \min_{𝐰\in 𝒳,\;\eta \in \mathbb{R},\;\Theta }\; & \rho B+\sum_{r\in ℛ}\sum_{i\in 𝒩}c_{r,i}^{pre}x_{r,i}\;+\;\beta \;\eta \;+\;\sum_{s\in \Omega_{S}}\Theta_{s}\\ \text{s.t.}\; & \text{(2), (3), and (35) for every cut generated so far,} \end{array}$$ (36)

with the spare-equipment components of 𝐱 integer. The integrality is handled by branch-and-Benders-cut: the master is solved by branch and bound, and cuts (35) are separated at integer-feasible incumbent nodes, so that the cut pool remains globally valid and no integer solution is cut off. Writing ${LB}^{\left(\nu \right)}$ for the optimal value of (36) at iteration ν and


$$\begin{array}{c} {UB}^{\left(\nu \right)}\;=\;\min_{\mu \leq \nu }\;[\rho {\hat{B}}^{\left(\mu \right)}+\sum_{r,i}c_{r,i}^{pre}{\hat{x}}_{r,i}^{\left(\mu \right)}+\beta {\hat{\eta }}^{\left(\mu \right)}+\sum_{s\in \Omega_{S}}Q_{s}({\hat{𝐰}}^{\left(\mu \right)},{\hat{\eta }}^{\left(\mu \right)})], \end{array}$$ (37)

the algorithm terminates when $({UB}^{\left(\nu \right)}-{LB}^{\left(\nu \right)})/\max\{1,|{UB}^{\left(\nu \right)}|\}\leq \epsilon$ with $\epsilon =10^{-4}$. Since $Q_{s}$ is convex and the cut pool is exact at every visited incumbent, the sequence ${LB}^{\left(\nu \right)}$ is non-decreasing and finite convergence follows from the finiteness of the set of dual bases of (33). The decomposition changes neither the model nor its optimum; it solves the same sample average approximation more efficiently when the scenario count is high, and it composes naturally with the replication scheme because subproblems are independent across scenarios. The procedure is stated in Algorithm 1.

**Algorithm 1 L-shaped solution of the risk-averse preparedness program**

1:  **input** reduced set $\Omega_{S}$ with probabilities $\pi_{s}$; parameters $\alpha ,\beta ,\rho ,B^{max}$; tolerance ϵ

2:  initialize cut pool 𝒞←∅, UB←+∞, $\Theta_{s}\geq \Theta_{s}^{lo}$, ν←0

3:  **repeat**

4:   ν←ν+1

5:   solve master (36) with cut pool 𝒞 by branch and bound → $\left(\hat{𝐰},\hat{\eta },\hat{\Theta }\right)$, LB← master value

6:   **for all** $s\in \Omega_{S}$ **in parallel do**

7:    solve subproblem (33) at $\left(\hat{𝐰},\hat{\eta }\right)$ → value $Q_{s}$ and optimal duals ${\hat{\lambda }}_{s}$

8:    **if** $Q_{s}>{\hat{\Theta }}_{s}+\epsilon_{cut}$ **then** add cut (35) to 𝒞

9:    **end if**

10:   **end for**

11:   $UB\leftarrow \min\{UB,\;\rho \hat{B}+\sum_{r,i}c_{r,i}^{pre}{\hat{x}}_{r,i}+\beta \hat{\eta }+\sum_{s}Q_{s}\}$       ▷ Eq. (37)

12:  **until** (UB−LB)/max{1,|UB|}≤ϵ

13:  **return** incumbent $\left(\hat{B},\hat{𝐱}\right)$ attaining UB, together with ${CVaR}_{\alpha }$ and EUE

The selection of the risk parameters governs how conservatively the reserve is sized: the confidence level α fixes which fraction of scenarios constitutes the tail, and the weight β or the tolerance Γ in (26) sets how heavily that tail is penalized, so together they translate a qualitative risk appetite into a quantitative preparedness posture. Rather than asserting a single correct value, the methodology treats these as levers whose effect is examined by re-solving (28) across a range of α and β and tracing how the optimal reserve fund scale and the composition of the pre-positioned resources respond to increasing risk aversion. The out-of-sample evaluation (31) plays a role beyond bounding the gap: it guards against the optimism inherent in any sample-based optimization, since a plan that performs excellently on the reduced set used to construct it may perform worse on fresh scenarios. Only when in-sample and out-of-sample costs are close, and the first-stage decisions are stable across replications, can the utility trust that the recommended reserve and resource configuration reflect the underlying risk rather than the idiosyncrasies of one sample. This validation discipline matters particularly here, because the conditional value-at-risk depends on the tail, which is the part of the distribution most sensitive to sampling, and it is why the termination criterion in (32) is framed by statistical stability rather than by a fixed scenario budget.

All computations reported below were performed on a workstation with an Apple M4 processor (10 cores) and 16 GB of memory under macOS 26.5, using Python 3.9.6 with NumPy 2.0.2 and SciPy 1.13.1, whose linear and mixed-integer programming interface is built on the HiGHS solver. Linear and mixed-integer subproblems were solved with a relative optimality tolerance of 10^–4^ and default presolve; no time limit was reached. Replications were executed sequentially and each instance was solved single-threaded, so the reported wall-clock times contain no parallel speed-up and are directly comparable across methods. Scenario generation and reduction are timed separately from optimization and are excluded from the solver timings; generating and reducing one batch of 10^4^ raw scenarios to 200 representatives takes 0.54 s under this configuration. Random seeds are fixed and stated in Table 2, so that every reported quantity derives from a specified draw.

**Table 2:** Case-study parameters, generation settings and random seeds.

Group	Symbol	Value	Basis / unit
System	|𝒩|	118	service nodes, IEEE 118-bus geometry
System	zone split	46/40/32	urban / suburban / rural nodes
System	nodal peak load	𝒰(55,120) / 𝒰(30,80) / 𝒰(12,45)	MW, by zone
System	total load	7237	MW
System	critical nodes	17	15% of nodes, drawn without replacement
System	depots	12	nodes drawn without replacement
System	|𝒯|, Δt	24, 1	periods, h
Value of lost load	$v_{i,0}^{lol}$ critical	$𝒰(28,52)\times 10^{3}$	$/MWh at *t* = 0
Value of lost load	$v_{i,0}^{lol}$ ordinary	$𝒰(6,14)\times 10^{3}$	$/MWh at *t* = 0
Value of lost load	γ	0.06	$v_{i,t}^{lol}=v_{i,0}^{lol}(1+\gamma t)$
Cost	$c^{pre}$ crew / mat. / equip.	0.15 / 0.02 / 1.05	$M per unit
Cost	$d^{dis}$ crew / mat. / equip.	0.010 / 0.004 / 0.030	$M per unit deployed
Cost	$e^{buy}$ crew / mat. / equip.	0.42 / 0.055 / 2.10	$M per unit, post-event premium
Cost	ρ	0.06	holding rate over the horizon
Cost	$B^{max}$	450	$M
Capability	κ crew / equip. / mat.	3.0 / 5.5 / 4.0	MW per unit per period (mat.: cumulative)
Capability	$\chi_{k}$	0.12 / 0.35 / 0.95	equipment coverage of ordinary load, by cause
Capability	$a_{k,t}$ crews	min(0.12+0.023t,1) / 0.95 / 0.08	reachability, disaster / failure / attack
Capability	$a_{k,t}^{en}$ equipment	min(0.40+0.018t,1) / 0.85 / 0.95	reachability, disaster / failure / attack
Capability	$L^{lead}$	2	periods, emergency procurement delivery
Capability	$\overset{―}{Q}$	0.45	fraction of staged material capacity
Risk	α, β	0.95, 1.0	baseline confidence level and weight
Scenario	|Ω|, $N^{red}$, $N^{\prime }$	10000, 200, 5000	raw / retained / validation
Scenario	$p_{k}$	0.20 / 0.62 / 0.18	disaster / failure / attack
Scenario	$\lambda_{1},\lambda_{2}$	1.6, 1.3	tail weights on total and peak loss in (20)
SAA	*M*, ϵ	30, 10^–4^	replications, relative tolerance
Seeds	system / raw / reduction / validation	20240608 / 20240609 / 20240610 / 20240707	
Seeds	supplementary analyses	20260829	base seed, offsets stated in text

## Case studies

### Case description

**Fig 3:**
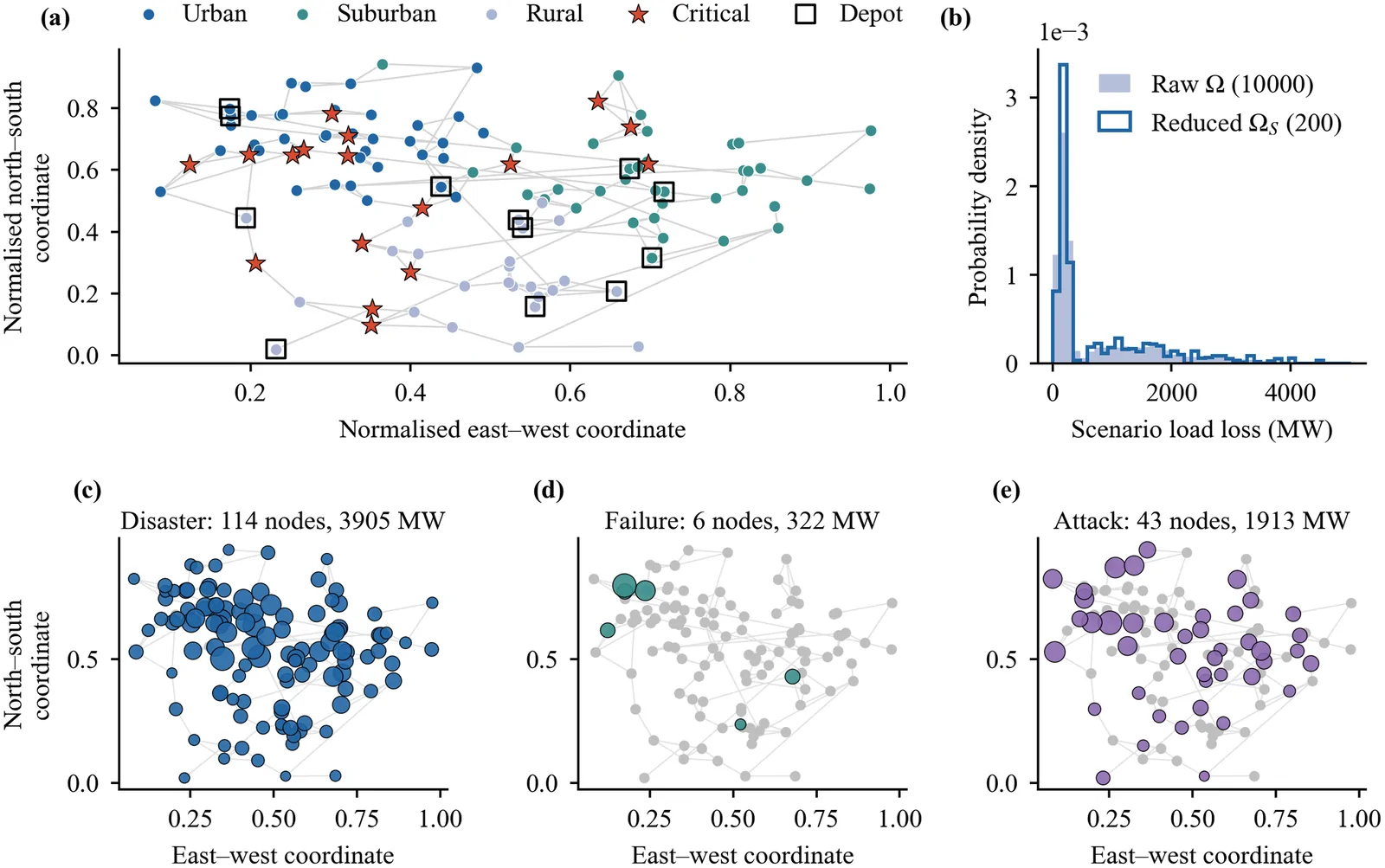
Test system and multi-type scenario set. (a) The 118-node service network with depots and critical nodes. (b) The loss distribution of the raw versus reduced scenario sets. (c)–(e) Representative damage footprints of the three cause types.

The proposed model is examined on a service territory abstracted from a transmission-level utility, at a scale chosen to reflect the spatial and temporal complexity of wide-area blackout preparedness rather than an illustrative toy. The network contains 118 service nodes whose geometry is anchored on the standard IEEE 118-bus topology distributed with the MATPOWER package [44] and is partitioned into urban, suburban and rural zones, with an aggregate peak demand of 7237 MW; seventeen nodes serving hospitals, data centres and transport hubs are designated as critical and carry a higher value of lost load that grows with outage duration. The post-event response is resolved over a 24-hour restoration horizon, and the emergency portfolio contains the three resource classes of the Mathematical formulation section. The network, its critical nodes, and the twelve depots at which resources are pre-positioned are shown in panel (a) of Fig 3, which makes explicit that the preparedness decision is spatial as well as financial.

The role of the IEEE topology should be stated precisely, because it is narrower than the label might suggest. The instance is a synthetic planning instance that borrows the bus count and relative geometry of the IEEE 118-bus case as a spatial scaffold; the geographic coordinates, urban–suburban–rural zoning, nodal peak loads, criticality designations and depot sites are generated by the procedure documented in Table 2 and are not part of the MATPOWER dataset. No admittance, line rating, generation limit or other electrical datum from that case enters the optimization, and no power-flow feasibility condition is imposed. The topology therefore supplies the spatial structure over which hazard footprints are drawn and reachability is defined, and the case study should be read as a stylized planning experiment rather than as validation against an operational power-system model.

Every parameter needed to reproduce the study is given in Table 2, together with its unit, the random seed that fixes it and the basis on which it was chosen. Values were set to the order of magnitude reported for transmission utilities in the resilience-planning literature rather than calibrated to a particular company: spare equipment is the costly but decisive instrument for re-energizing critical load, crews are the versatile workhorse of bulk physical repair, the reserve carries a six-percent holding cost representing the opportunity cost of committed liquid capital, and emergency procurement is priced at a premium reflecting a strained post-event market. The value of lost load is drawn from ranges consistent with published interruption-cost estimates for critical and ordinary customers, and its escalation with outage duration is imposed through the linear factor 1+γt; since the analysis below shows this parameter to be the dominant economic driver, its range is treated as an object of sensitivity analysis rather than as a calibrated constant.

The multi-type uncertainty is generated by Monte Carlo sampling over the three cause types, a standard basis for power-system reliability evaluation under uncertainty [45], and each type carries a distinct spatial signature: natural disasters produce wide-area spatially correlated damage; equipment failures strike a few localized nodes; and cyber attacks disable a dispersed set of nodes whose restoration depends on backup equipment rather than physical repair. These signatures are contrasted in panels (c) to (e) of Fig 3, where a representative disaster affects 114 nodes for 3905 MW of lost load, a failure affects six nodes for 322 MW, and an attack affects 43 dispersed nodes for 1913 MW. A raw set of 10,000 scenarios is condensed by scenario reduction into 200 representative scenarios, whose loss distribution, overlaid on that of the raw set in panel (b) of Fig 3, preserves the heavy upper tail on which the risk measure depends; a further 5000 independent scenarios are reserved for out-of-sample validation.

The generative model behind these signatures is specified in Table 3 so that the scenario set can be reproduced independently. Cause occurrence is a categorical draw with the probabilities of Table 2, and the three conditional damage models differ in footprint mechanism rather than in a shared severity scale. Disasters place an epicentre uniformly over the node set, draw a decay radius and a severity multiplier, and apply an isotropic Gaussian attenuation of the nodal load, with an independent Bernoulli survival mask that leaves a small fraction of nodes within the footprint unaffected. Equipment failures draw a small number of nodes uniformly without replacement and remove a large fraction of their load. Cyber attacks draw a much larger and spatially uncorrelated node set and remove a comparable fraction of load, which reproduces the dispersed footprint characteristic of a control-layer compromise. Within a scenario, the nodal losses generated by a disaster are spatially correlated through the common epicentre and radius, whereas those generated by failures and attacks are conditionally independent across nodes; across scenarios, draws are independent, and cause types are mutually exclusive as discussed after (17).

**Table 3:** Conditional damage model of each cause type.

Cause	Footprint mechanism	Nodal loss $D_{i}$
Disaster	epicentre ~ uniform over 𝒩; radius R~𝒰(0.22,0.50)	$D_{i}=\delta \;e^{-(\|{𝐩}_{i}-{𝐩}_{0}\|/R{)}^{2}}\;b_{i}\;P_{i}$
	severity δ~𝒰(0.55,1.00); mask $b_{i}\sim Bern(0.94)$	spatially correlated, wide area
Failure	m~𝒰{2,...,6} nodes without replacement	$D_{i}=\varphi_{i}P_{i}$, $\varphi_{i}\sim 𝒰(0.50,1.00)$
		localized, few nodes
Attack	m~𝒰{14,...,43} nodes without replacement	$D_{i}=\varphi_{i}P_{i}$, $\varphi_{i}\sim 𝒰(0.45,1.00)$
		dispersed, spatially uncorrelated

Each instance is solved by the sample-average-approximation scheme of the Mathematical formulation section, with thirty independent replications forming the statistical bounds, and every returned plan is evaluated on the independent validation set to confirm its out-of-sample performance.

The four benchmarks against which the proposed model is measured are defined algorithmically here, since their construction determines what the comparison establishes. The decoupled scheme fixes the reserve exogenously before allocating resources, mimicking a utility that sets its emergency fund by a financial rule and only then asks what it can buy: the reserve is set to $B^{dec}=\min\{B^{max},\;0.06\;{𝔼}_{\pi }[L_{s}^{0}]+35\}$ $M, where $L_{s}^{0}$ is the loss incurred in scenario *s* with no resources staged, which yields 62.5 $M for the baseline instance; the resource allocation is then optimized exactly as in the proposed model with *B* held at that value, so the two schemes differ only in whether the reserve is endogenous. The risk-neutral scheme solves the identical program with β=0, retaining the coupling but pricing only the expected loss. The single-hazard scheme optimizes on the disaster-only subset of the reduced set, with probabilities renormalized to sum to one, and is then evaluated on the full multi-type validation set, which represents a utility that has calibrated its preparedness to its dominant historical hazard. The deterministic expected-value scheme replaces the scenario set with the single probability-weighted mean loss vector $\bar{𝐃}=\sum_{s}\pi_{s}{𝐃}_{s}$, optimizes against that one scenario with β=0, and is likewise evaluated on the full validation set. All five plans are evaluated by the same out-of-sample procedure with identical parameters, so that differences in reported performance are attributable to the first-stage decisions alone.

### Experimental results analysis

The preparedness plan returned by the proposed model under the baseline parameters provides the reference against which every benchmark is measured, and its structure already shows the value of treating the reserve as an endogenous decision. As reported in Table 4, the model commits a reserve fund of $B^{*}=164.5$ $M, of which 159.2 $M is converted into pre-positioned resources, namely 762 crew units, 886 units of materials and 26 items of spare equipment. The preparation cost of (1) is accordingly $C^{pre}=159.16+\rho B^{*}=159.16+9.87=169.03$ $M, and the unconverted residual of 164.50−159.16=5.34 $M remains liquid to finance emergency procurement under (16) without itself appearing as an expense; the earlier description of this decomposition was imprecise and has been corrected. The allocation splits the staged budget as 0.72/0.11/0.17 among crews, materials and equipment, and this balance is enforced internally by the funds–resources coupling of (2) rather than imposed from outside: the reserve sizes the resources, and the resources, through the recourse of (4)–(21), size the loss. This is exactly the interdependence that a sequential treatment severs, and the comparison that follows quantifies the cost of severing it.

**Table 4:** First-stage plan of the proposed versus decoupled scheme.

Quantity	Proposed	Decoupled
Reserve fund $B^{*}$ ($M)	164.5	62.5
Crews (units / $M)	762 / 114.3	251 / 37.7
Materials (units / $M)	886 / 17.7	829 / 16.6
Spare equip. (units / $M)	26 / 27.2	8 / 8.2
Allocation share (crew/mat/equip)	0.72/0.11/0.17	0.60/0.27/0.13

**Fig 4:**
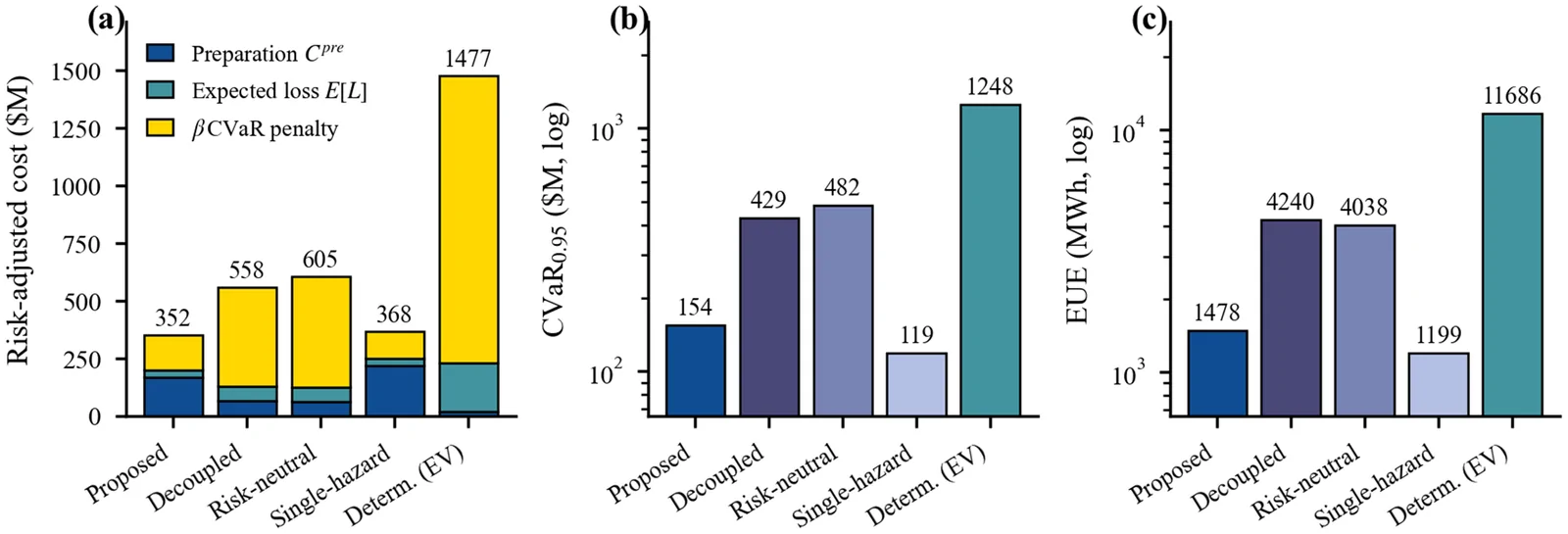
Out-of-sample comparison of the five preparedness schemes. (a) Risk-adjusted cost composition. (b) Conditional value-at-risk. (c) Expected unserved energy.

The central comparison places the proposed coordinated risk-averse model against the four benchmarks defined above, each of which relaxes one of its features. All plans are evaluated out of sample on the 5000 validation scenarios, and their cost composition, tail risk and reliability are reported in Fig 4, while Table 5 lists the underlying figures together with the reserve scale $B^{*}$. The proposed model attains the lowest risk-adjusted total cost, 352.1 $M, against 558.2 and 604.7 $M for the decoupled and risk-neutral schemes and 1476.7 $M for the deterministic one. The mechanism behind this ranking is visible in the cost composition of panel (a): the deterministic and risk-neutral plans under-provision the reserve, at $B^{*}=17.8$ and 63.7 $M, so their small preparation outlay is overwhelmed by an expected second-stage loss of 209.8 and 59.7 $M and, above all, by a large tail penalty, whereas the proposed plan spends more at the front to suppress exactly that tail.

**Table 5:** Out-of-sample performance of the five methods on the primary validation set.

Method	$B^{*}$ ($M)	Total ($M)	$C^{pre}$ ($M)	CVaR_0.95_ ($M)	EUE (MWh)	$\hat{\Delta }$ (%)
Proposed	164.5	352.1	169.0	154.3	1478	−1.01
Decoupled	62.5	558.2	66.2	428.5	4240	−1.38
Risk-neutral	63.7	604.7	63.2	481.8	4038	−2.58
Single-hazard	206.7	368.2	219.1	119.3	1199	−0.41
Determ. (EV)	17.8	1476.7	18.7	1248.2	11686	−1.68

The last column of Table 5 requires an explicit definition, because the corresponding column of the earlier version was mislabelled. The quantity $\hat{\Delta }$ is the relative difference between the out-of-sample and the in-sample reduced-set objective of one and the same plan, $\hat{\Delta }=({\hat{U}}_{N^{\prime }}-{\hat{v}}_{N}^{red})/{\hat{U}}_{N^{\prime }}$. It is a diagnostic of the agreement between the two evaluations of a fixed policy, and it is not an optimality gap; nothing prevents it from being negative, and the fact that it is negative for all five methods says that the tail-weighted reduced set is mildly conservative, so the plan performs slightly better on fresh scenarios than the set used to construct it predicted. An optimality gap, which by construction is non-negative up to sampling error, requires the lower-bound estimator of (29) and is reported separately in Table 8.

The tail and reliability panels of Fig 4 sharpen this reading and expose the limits of the partial benchmarks. Panel (b) shows that the proposed model holds the conditional value-at-risk to ${CVaR}_{0.95}=154.3$ $M, well below the 428.5 and 481.8 $M borne by the decoupled and risk-neutral schemes and far below the 1248.2 $M of the deterministic plan, which follows directly from pricing worst-case losses through the CVaR term of (24)–(25) rather than their average alone. Panel (c) tells the same story in physical units: the expected unserved energy of (27) falls to 1478 MWh for the proposed plan, against 4038–4240 MWh for the decoupled and risk-neutral schemes and 11,686 MWh for the deterministic one. The single-hazard scheme is the instructive exception. By preparing only for disasters it reaches a lower tail of 119.3 $M and unserved energy of 1199 MWh, but it does so by over-sizing the reserve to $B^{*}=206.7$ $M and inflating the preparation cost to 219.1 $M, so that its total cost of 368.2 $M still exceeds that of the proposed model. The lesson for a utility is that tuning preparedness to the single most severe hazard is safe but wasteful, whereas the multi-type formulation reaches comparable protection at a materially lower cost by hedging the whole spectrum of causes at once; the divergence in first-stage plans is quantified in Table 4, where the reserve-starved decoupled scheme stages roughly a third of the crews and equipment of the proposed one.

Point estimates on a single validation set are not sufficient evidence for a stochastic simulation study, so each of the five fixed first-stage plans was re-evaluated on twenty independently drawn validation sets of 5000 scenarios each. Table 6 reports the resulting means with 95% confidence half-widths. The ordering obtained on the primary validation set is reproduced on all three metrics, and the intervals of the proposed plan are disjoint from those of the decoupled, risk-neutral and deterministic schemes by a wide margin, so the ranking is not an artefact of one draw. The single-hazard scheme again attains a lower tail and lower unserved energy than the proposed plan, and the separation is statistically clear rather than marginal; its disadvantage lies entirely in the preparation cost it pays for that protection, which is the trade-off the total-cost column captures.

**Table 6:** Out-of-sample performance over twenty independent validation sets. Entries are the mean ± the 95% confidence half-width.

Method	Total ($M)	CVaR_0.95_ ($M)	EUE (MWh)
Proposed	356.3±1.8	157.6±1.6	1557±26
Decoupled	567.7±5.2	435.0±4.4	4453±67
Risk-neutral	618.9±6.5	493.1±5.7	4242±70
Single-hazard	370.9±1.4	121.3±1.2	1264±20
Determ. (EV)	1507.7±10.7	1270.0±8.3	12197±167

### Fidelity of the scenario reduction

**Fig 5:**
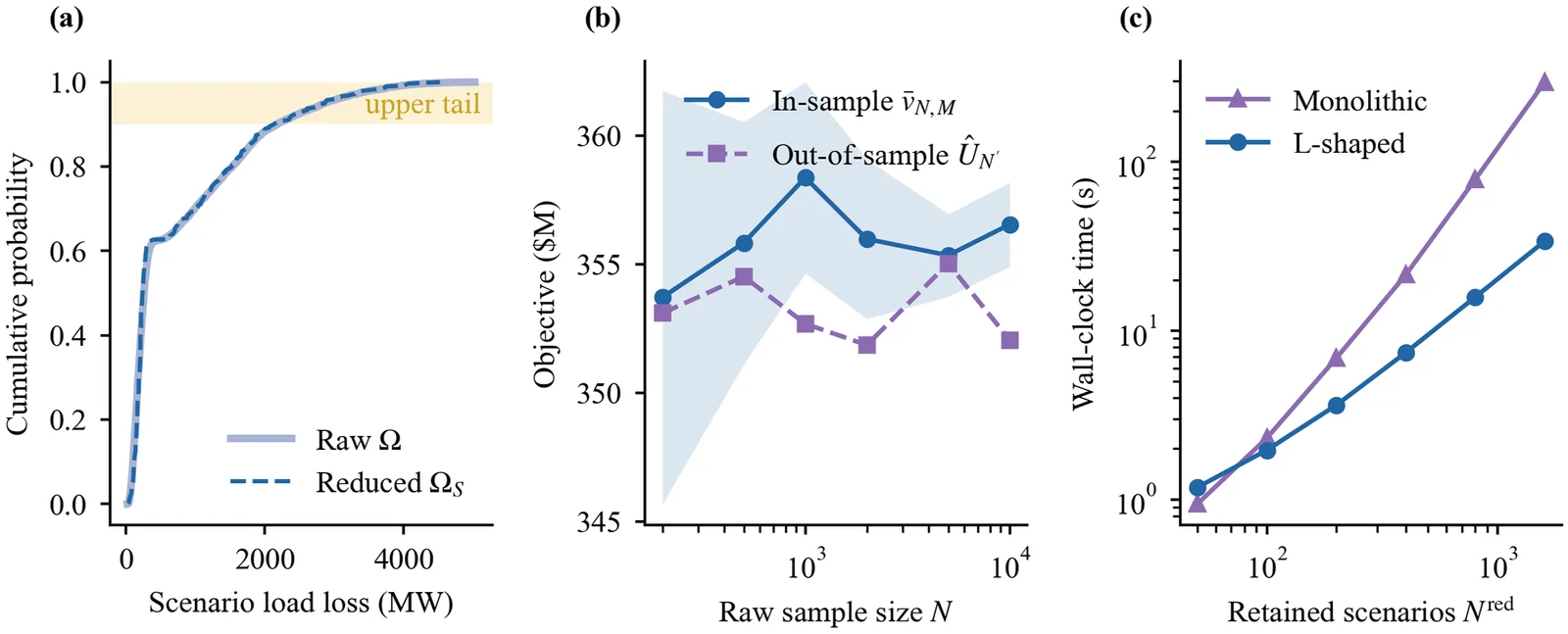
Reduction fidelity, SAA convergence and computational scaling. (a) Reduction fidelity via the loss-distribution ECDF. (b) SAA convergence of the in-sample and out-of-sample estimates. (c) Wall-clock scaling of the monolithic solver versus the L-shaped decomposition on the node-resolved formulation.

**Fig 6:**
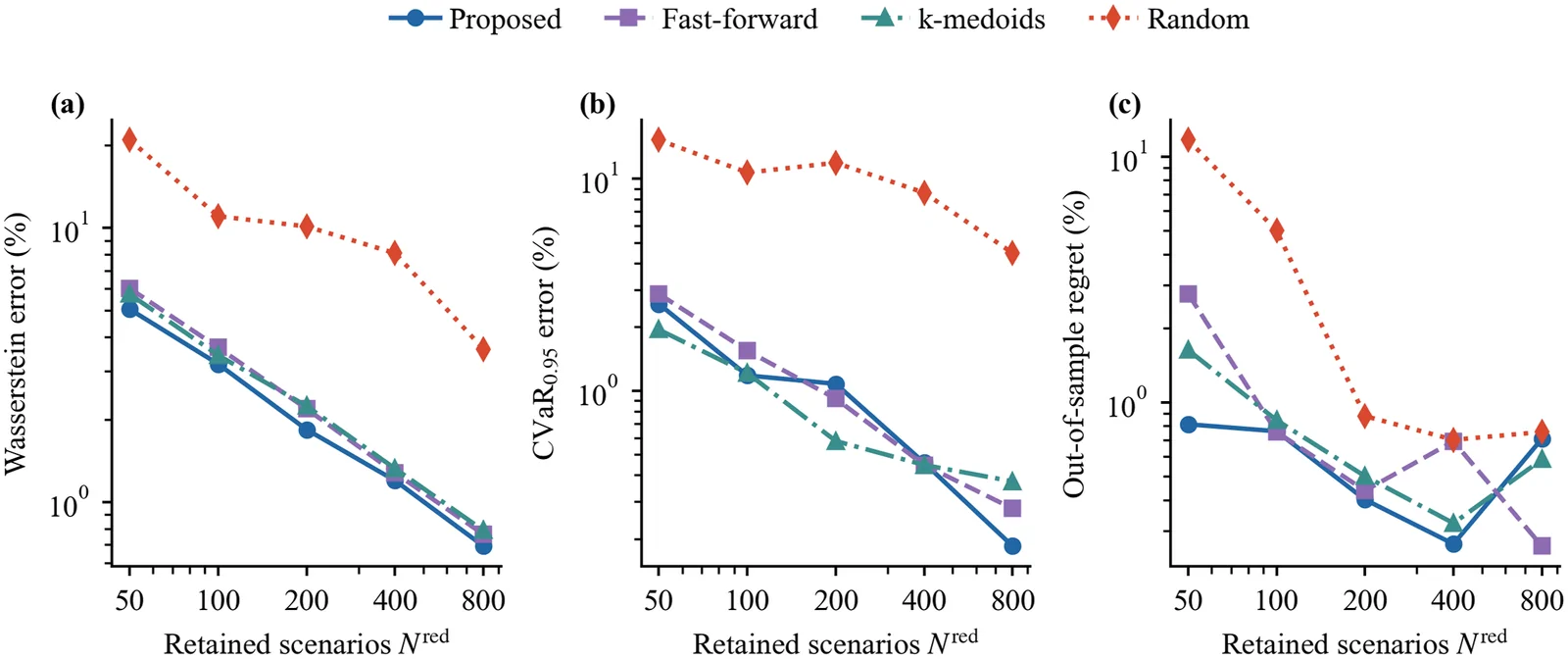
Comparison of scenario-reduction schemes against the raw empirical loss distribution. (a) Wasserstein-1 error. (b) CVaR_0.95_ error. (c) Out-of-sample regret of the resulting first-stage plan.

The fidelity of the scenario reduction supporting these results is examined first through the empirical cumulative distribution in panel (a) of Fig 5, which overlays the reduced set on the raw set. The two curves are close over the whole support, including the upper-tail band that the conditional value-at-risk integrates, and the reduction shrinks the problem by a factor of fifty. Visual agreement of cumulative distributions is nevertheless weak evidence for tail preservation, since the tail occupies a small share of the horizontal axis and large relative errors there are hard to see. The reduction was therefore assessed quantitatively against three alternatives on identical raw samples of 3000 scenarios, repeated over four independent draws: random subsampling with uniform weights, medoid clustering in the same attribute space but without the tail weights $\lambda_{1},\lambda_{2}$ of (20), and Dupačová fast-forward selection with optimal redistribution. Four error measures are reported against the raw empirical distribution, namely the Wasserstein-1 distance normalized by the mean loss, and the relative errors in VaR_0.95_, CVaR_0.95_ and the 0.99-quantile, together with a decision-quality measure, the out-of-sample regret of the first-stage plan obtained from the reduced set relative to the plan obtained from the full raw set. Results appear in Table 7 and Fig 6.

**Table 7:** Tail-fidelity and decision-quality errors of four scenario-reduction schemes, measured against the raw empirical loss distribution. All entries are percentages, averaged over four independent raw samples of 3000 scenarios.

Scheme	$N^{red}$	*W*_1_ error	VaR_0.95_ error	CVaR_0.95_ error	*q*_0.99_ error	Regret
Proposed (tail-weighted)	50	5.05	5.31	2.57	9.70	0.81
	200	1.84	5.23	1.08	2.67	0.40
	800	0.69	0.88	0.18	0.41	0.71
Fast-forward selection	50	6.02	9.38	2.86	5.45	2.76
	200	2.19	1.69	0.92	3.79	0.44
	800	0.76	1.23	0.28	0.33	0.26
*k*-medoids (no tail weights)	50	5.73	8.82	1.94	5.54	1.63
	200	2.24	4.97	0.58	1.23	0.50
	800	0.79	0.90	0.37	0.43	0.58
Random subsampling	50	20.92	29.37	15.25	16.82	11.67
	200	10.13	13.05	11.91	10.18	0.88
	800	3.62	5.69	4.46	3.79	0.76

Three findings follow. First, the gap between any clustering- or selection-based reduction and random subsampling is large and persists across retained-set sizes: at the baseline size of 200 representatives, random subsampling misstates CVaR_0.95_ by 11.91% and the 0.99-quantile by 10.18%, against 1.08% and 2.67% for the proposed scheme, and its Wasserstein error remains above that of the proposed scheme even at 800 representatives. This is the quantitative content of the claim that a tail-preserving reduction is needed rather than merely convenient. Second, the proposed scheme attains the lowest Wasserstein error at every size tested and the lowest CVaR error at the larger sizes, but its advantage over fast-forward selection and over plain medoid clustering is modest and not uniform across measures: fast-forward selection recovers VaR_0.95_ more accurately at 200 representatives, and plain clustering attains a lower CVaR error at that size. The tail weights therefore buy a consistent improvement in the aggregate distance and in the extreme quantile, not a dominance across all tail statistics, and the honest reading is that any of the three structured schemes would support the conclusions of this paper whereas random subsampling would not. Third, the decision-quality column behaves less smoothly than the distributional columns, with regret at 800 representatives occasionally exceeding that at 400 for the same scheme. This reflects the flatness of the first-stage objective near its optimum, which is examined further in the joint-uncertainty analysis below and which is itself a finding of practical relevance: the reserve is weakly identified by the data, so the reduction should be judged on the cost consequence of its output rather than on the recovered value of *B* alone.

### Statistical validation and computational performance

The convergence of the sample-average-approximation scheme is reported in panel (b) of Fig 5. As the sample size *N* grows from 200 to 10,000, the in-sample and out-of-sample estimates stabilize around 355 $M and their separation closes to within the statistical error, while the standard error of the lower-bound estimator contracts from 4.1 to 0.8 $M.

The statistical claims attached to this convergence are stated with the estimators of (29)–(32) rather than with the reduced-set objective, because the reduced set is not an ordinary independent sample. Table 7 of the previous subsection concerns distributional fidelity; Table 8 concerns the statistical quality of the solution. For each raw sample size, thirty independent samples were solved without reduction to form the lower-bound estimator ${\bar{L}}_{N,M}^{raw}$, the same thirty samples were reduced and solved to form ${\bar{L}}_{N,M}^{red}$ and hence the reduction bias ${\hat{b}}_{N}$ of (30), and the candidate plan was evaluated on twenty independent validation sets to form ${\hat{U}}_{N^{\prime }}$ with its own standard error.

**Table 8:** Sample-average-approximation statistics with a lower-bound estimator constructed on unreduced samples. Settings: *M* = 30 replications, $N^{\prime }=5000$, twenty validation sets.

*N*	${\bar{L}}_{N,M}^{raw}$ ($M)	${\hat{b}}_{N}$ ($M)	${\hat{U}}_{N^{\prime }}$ ($M)	${\hat{gap}}_{N}$ ($M)	${\hat{gap}}_{N}$ (%)
200	351.86±4.36	−0.43	359.67±1.32	7.80±8.93	2.17
500	352.67±2.45	−0.50	358.60±0.90	5.93±5.11	1.65
1000	354.79±1.39	−0.80	361.88±0.77	7.09±3.12	1.96
2000	357.40±1.46	−0.02	356.72±1.33	−0.68±3.88	−0.19

The gaps computed against a valid lower bound are non-negative at every sample size except the largest, where the point estimate is −0.68 $M with a 95% half-width of 3.88 $M and is therefore indistinguishable from zero; a negative point difference of this magnitude is the expected consequence of sampling error rather than evidence of a sign error, and the interval is reported so that it can be read as such. The reduction bias ${\hat{b}}_{N}$ is negative and small throughout, at most 0.80 $M or 0.22% of the objective, which indicates that the tail-weighted reduced set yields a marginally lower in-sample optimum than the corresponding unreduced sample rather than a materially different one.

**Fig 7:**
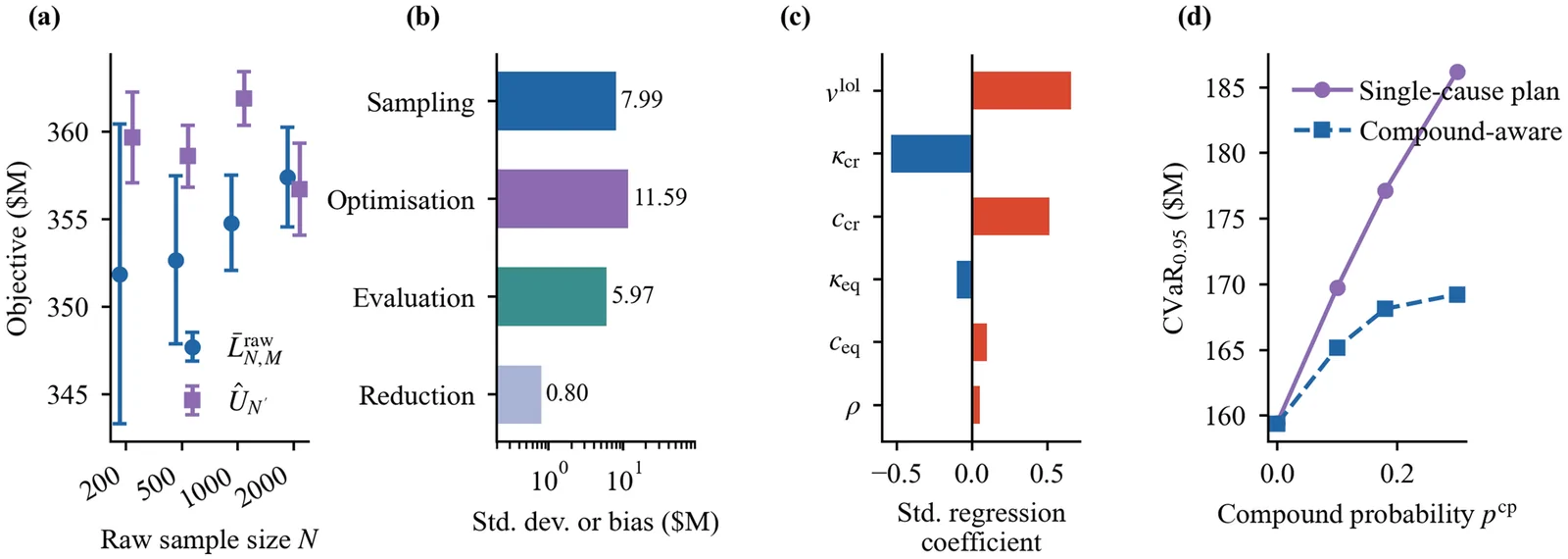
Statistical structure and robustness of the solution. (a) Lower-bound and out-of-sample estimators with 95% intervals. (b) Decomposition of the four error sources. (c) Standardized regression coefficients of the total cost under joint parameter uncertainty. (d) Tail risk of the single-cause and compound-aware plans as the compound-event probability varies.

Separating the four error sources defined in the Mathematical formulation section gives the picture in panel (b) of Fig 7, and it changes what the narrow confidence intervals of Table 8 should be taken to mean. Measured as standard deviations on the objective at the baseline setting, Monte Carlo sampling contributes 7.99 $M across independent raw samples, out-of-sample evaluation contributes 5.97 $M across independent validation sets, and scenario reduction contributes a systematic component of at most 0.80 $M. The largest single component is optimization error: the dispersion of the returned objective across independent single starts of the first-stage search on one fixed instance is 11.59 $M, which exceeds both sampling terms. The practical implication is that multi-start search is not a refinement but a requirement here, and that reporting a sampling confidence interval alone would understate the uncertainty attached to any single reported plan. It also explains why the reduction, despite being the component most often suspected, is not the binding source of error in this problem.

Panel (c) of Fig 5 contrasts the wall-clock time of solving the node-resolved program (1)–(27) monolithically with that of the L-shaped decomposition of Algorithm 1 on identical instances, under the environment disclosed in the Mathematical formulation section. As the retained-scenario count rises from 50 to 1600, the monolithic solve time grows from 0.94 to 296.3 s while the decomposition grows from 1.18 to 33.9 s, requiring between 11 and 24 iterations, and the two approaches agree on the optimal value to within a relative difference of $2.6\times 10^{-5}$, confirming that the decomposition changes the solution method and not the model. The decomposition is slower than the monolithic solver on the smallest instance, where the overhead of repeated master solves is not amortized over enough subproblems, and its advantage grows with the scenario count, reaching a factor of 8.7 at the largest instance tested. These times refer to the node-resolved instances of (1)–(27), whereas the solve times reported later in the robustness analysis refer to the aggregated instances described at the end of the Mathematical formulation section on which the policy experiments are run; the two sets of timings measure different problems and are not comparable to one another.

### Restoration dynamics

**Fig 8:**
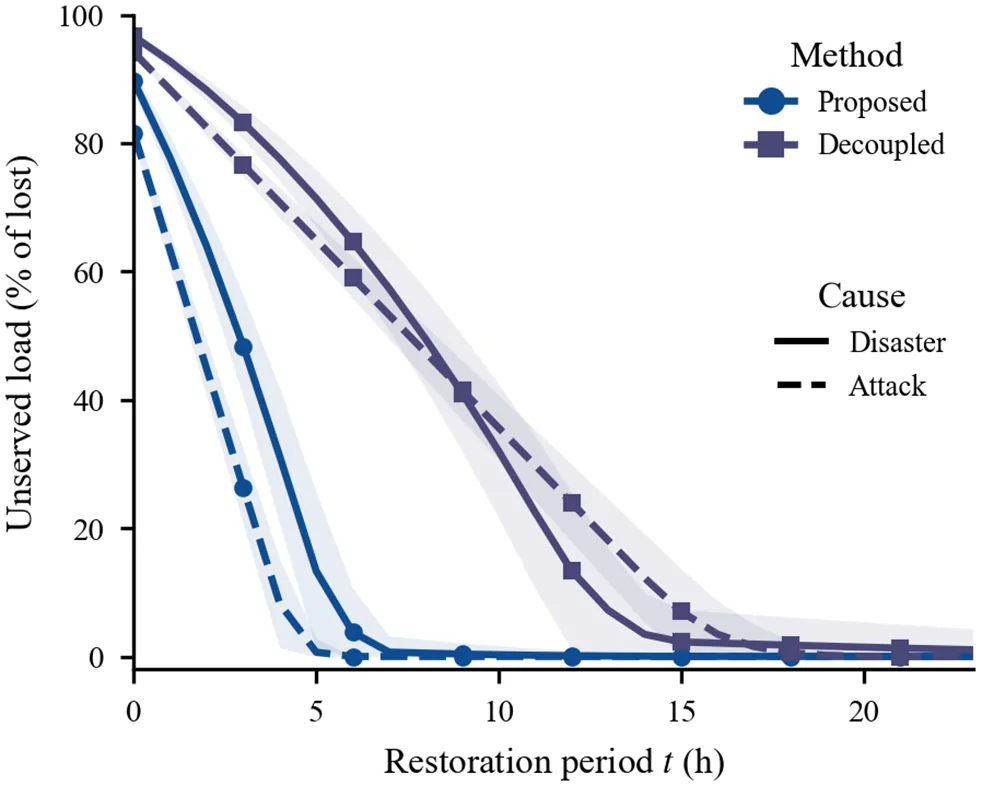
Restoration dynamics under disaster and cyber-attack scenarios. Unserved load over the restoration horizon for the proposed versus decoupled plans; shaded bands denote one standard deviation across scenarios.

The temporal mechanism by which the coordinated plan converts the reserve into avoided loss is displayed in Fig 8, which traces unserved load over the restoration horizon for the proposed and decoupled plans under the two large-scale cause types. Equipment failures are omitted, because they are localized and small and are restored within the first hour by any adequately resourced plan. For both disasters and cyber attacks, the proposed plan clears the outage substantially faster: under disaster scenarios it restores about ninety-five percent of the lost load within six hours, while the reserve-starved decoupled plan still leaves more than forty percent unserved at that point and does not approach full restoration until after the eighteenth hour. This gap is rooted in the recourse technology of (4)–(11): the proposed plan stages enough crews and materials to sustain a high per-period repair rate once reachability improves, whereas the decoupled plan, constrained by its small reserve, cannot. Because the value of lost load rises with outage duration through $v_{i,t}^{lol}$, this faster early restoration is exactly where avoided cost is concentrated, which is why a plan that restores the same energy earlier records a much smaller second-stage loss in (21). The absolute restoration speeds should be read subject to the aggregation and occupancy assumptions declared in the Mathematical formulation section, which omit travel and service durations and therefore describe an upper envelope; the comparison between the two plans is unaffected, since both are evaluated under the same technology.

### Sensitivity analysis

**Fig 9:**
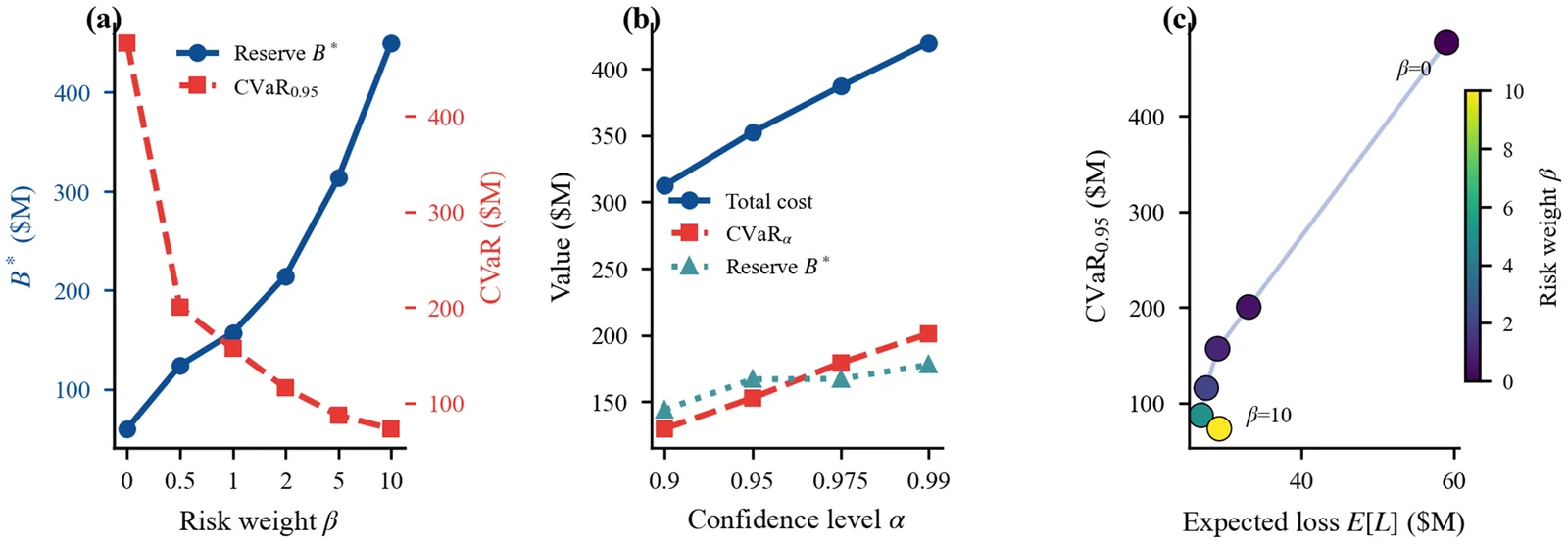
Sensitivity of the plan to the risk preference. (a) Reserve and tail risk versus the weight β. (b) Cost metrics versus the confidence level α. (c) Pareto frontier of expected loss against CVaR.

The risk-aversion weight β is the primary lever through which a utility translates its appetite for tail protection into a quantitative preparedness posture, and its effect is traced in panel (a) of Fig 9. As β increases from 0 to 10, the optimal reserve fund rises from 60.1 $M to the ceiling of 450.0 $M, while the conditional value-at-risk falls steeply from 477 to 73 $M; the expected loss is already near its floor and moves little, declining only from 59.0 to about 29 $M. This is the behaviour that the CVaR mechanism of (25) is designed to produce: weighting the tail more heavily makes the utility buy down extreme losses by enlarging the reserve and the resources it funds, at a rising marginal cost in committed capital. The trade-off is made explicit in the Pareto frontier of panel (c), which plots expected loss against tail risk as β varies and traces a smooth convex curve; a utility can read from it the reserve level at which further tail reduction ceases to be worth its capital cost, which turns an abstract risk preference into a defensible funding figure.

The confidence level α governs which fraction of scenarios constitutes the tail that the model controls, and its effect is shown in panel (b) of Fig 9. Tightening α from 0.90 to 0.99 shifts attention to ever rarer and more severe blackouts, so the optimal reserve grows from 144.0 to 177.7 $M, the conditional value-at-risk against which it is measured rises from 130 to 201 $M, and the total risk-adjusted cost increases from 312 to 420 $M. The upward movement of all three quantities reflects the structure of (23)–(24): a higher confidence level raises the threshold η and averages over a thinner and more extreme slice of the loss distribution, demanding a more conservative posture. For a decision-maker, α and β are therefore complementary dials, the former selecting how deep into the tail to look and the latter how heavily to weight what is found, and together they map a qualitative risk culture onto a concrete reserve and resource configuration.

**Fig 10:**
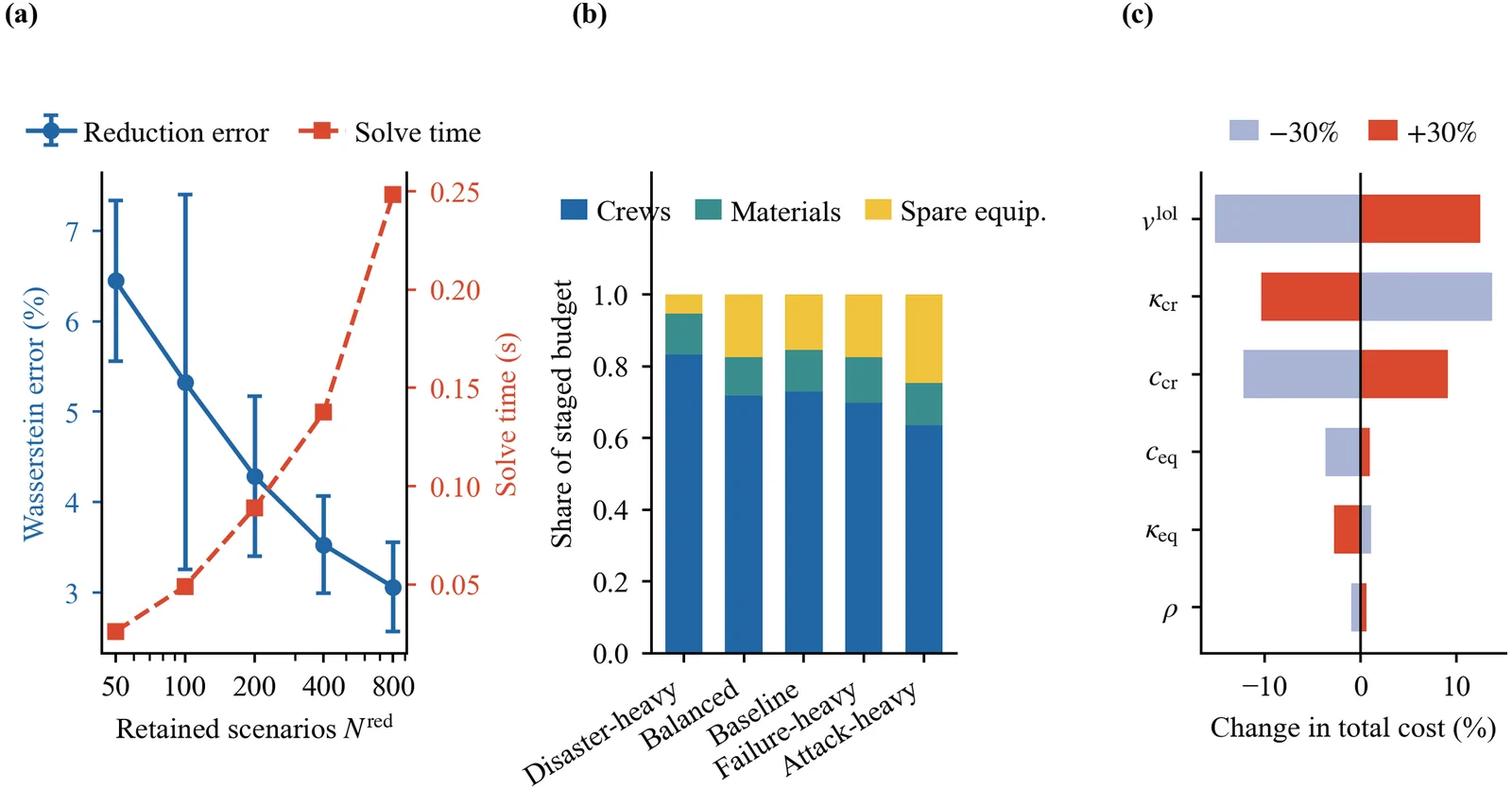
Robustness of the preparedness plan. (a) Reduction fidelity and solve time versus $N^{red}$. (b) Reserve allocation under varying cause mixes. (c) One-at-a-time ranking of economic parameters.

The interplay between scenario-reduction fidelity and computational effort is examined in panel (a) of Fig 10. As the retained scenario count $N^{red}$ increases from 50 to 800, the Wasserstein distance between the reduced-set and the out-of-sample loss distributions falls from 6.45% to 3.06% of the mean loss, while the solve time of the aggregated instance rises almost linearly from 0.026 to 0.248 s. The curve shows a clear diminishing return: the fidelity gained beyond a few hundred representatives is modest relative to its computational price, which justifies the baseline choice of $N^{red}=200$ as a balanced operating point that resolves the tail accurately while keeping each instance cheap enough to embed in the replication scheme of (29). The distance reported in this panel is measured against the out-of-sample empirical distribution, whereas Table 7 measures distances against the raw empirical distribution from which the reduced set is drawn; the two reference distributions are different and the corresponding values are therefore not interchangeable.

The robustness of the preparedness plan to the composition of the threat and to the economic environment is assessed in panels (b) and (c) of Fig 10, and both confirm that the model responds to the drivers a utility would expect. Panel (b) shows how the reserve allocation migrates as the cause mix varies: under a disaster-heavy mix the plan is dominated by crews and materials, the instruments of physical repair, and the equipment share falls to 0.05, whereas under an attack-heavy mix the share of spare equipment rises to 0.25, reflecting that cyber-induced outages are cleared through backup re-energization rather than crew repair. This response is generated by the cause-indexed technology of (6)–(9) rather than imposed: the productivity $\kappa_{r,k}$ and reachability $a_{k,t}$ of crews collapse in the attack regime while the coverage share $\chi_{k}$ of mobile equipment approaches unity, so the optimizer reallocates the staged budget towards equipment without any exogenous rule instructing it to do so. This sensitivity is a feature rather than a fragility: it shows that the coordinated model reshapes the resource portfolio to the prevailing hazard instead of applying a fixed rule of thumb. Panel (c) ranks the economic parameters by their effect on total cost under a ±30% one-at-a-time perturbation, and the value of lost load is the dominant driver, moving cost by −15.2% to +12.5%, followed by crew capability and cost; the reserve holding rate and the equipment parameters matter considerably less.

One-at-a-time perturbation holds every other parameter at its nominal value and therefore cannot detect interactions or report the joint uncertainty of the plan. The analysis was consequently repeated by perturbing the six economic and capability parameters simultaneously on a Latin hypercube of 120 draws over a ±30% range, re-optimizing at each draw and evaluating out of sample. The resulting risk-adjusted cost has a median of 347.6 $M with a 5th-to-95th-percentile range of 287.2 to 426.3 $M, and the crew share of the staged budget stays within 0.63 to 0.85, so the qualitative composition of the portfolio is stable under joint perturbation even though its scale is not. Standardized regression coefficients fitted to the 120 draws, shown in panel (c) of Fig 7, reproduce the one-at-a-time ranking and quantify it: the value of lost load carries a coefficient of 0.66, crew capability −0.53 and crew cost 0.52, while the equipment parameters and the holding rate remain below 0.11 in magnitude, and the regression explains 98% of the variance in total cost. The corresponding regression on the reserve scale explains only 27% of its variance, and the reserve itself ranges from 134.5 to 326.3 $M between the 5th and 95th percentiles. The contrast between the two regressions is the substantive finding of this analysis: the risk-adjusted cost is a well-determined function of the economic environment, whereas the reserve scale that achieves it is weakly identified, because the objective is flat in *B* near its optimum. A utility should therefore treat the model output as a defensible range for the reserve together with a sharp prescription for the resource composition, rather than as a single funding figure, and should direct measurement effort at the value of lost load and crew productivity, which is where the analytical return is concentrated.

The cost of the mutually exclusive single-cause assumption discussed after (17) was assessed by superimposing a second, milder cause on a fraction $p^{cp}$ of the scenarios, with nodal losses combined by saturating superposition so that no node loses more than its load. The baseline single-cause plan and a plan re-optimized on compound-aware scenarios were then evaluated on the same compound validation sets. As $p^{cp}$ rises from 0.10 to 0.30, the total cost of the single-cause plan rises from 369.5 to 388.1 $M and that of the compound-aware plan from 368.8 to 383.4 $M, so the regret of ignoring compound events grows from 0.20% to 1.21%. The effect on the tail is larger than the effect on the mean, as panel (d) of Fig 7 shows: at $p^{cp}=0.30$ the compound-aware plan holds CVaR_0.95_ to 169.2 $M against 186.2 $M for the single-cause plan. Two conclusions follow. The exclusivity assumption is not costless, and it costs most in precisely the tail region that motivates the model, so a utility operating in an environment with strong hazard interaction should extend the scenario process rather than rely on the present one. At the same time, the magnitude of the regret over the range tested is small relative to the differences between the preparedness schemes compared earlier, so the assumption does not overturn the comparative conclusions of this study; it bounds their precision rather than their direction.

## Conclusion

This paper addressed how a power utility should size its financial reserve and configure its emergency resources against large-scale blackouts of heterogeneous origin. A unified set of multi-type scenarios spanning natural disasters, equipment failures and cyber attacks was constructed and condensed by a tail-aware scenario reduction into a tractable representative set. On this basis, a two-stage risk-averse stochastic program was formulated to co-optimize the reserve fund scale and resource pre-positioning together with the post-event response, to bound extreme losses through a conditional-value-at-risk measure, and to be solved by sample average approximation with an L-shaped decomposition. The restoration technology separates a repair channel, in which crews and materials enter as complementary inputs, from a re-energization channel supplied by mobile equipment, and carries cause-dependent productivity and coverage in both, which is what allows the cause mix to reshape the resource portfolio endogenously.

Case studies on a stylized 118-node planning instance produced four quantitative results. The coordinated risk-averse plan attained an out-of-sample risk-adjusted cost of 352.1 $M against 558.2 $M for a decoupled scheme, 604.7 $M for a risk-neutral scheme and 1476.7 $M for a deterministic one, while holding CVaR_0.95_ to 154.3 $M and expected unserved energy to 1478 MWh; the ordering was reproduced with disjoint confidence intervals over twenty independent validation sets. The tail-aware reduction held the CVaR_0.95_ error to 1.08% and the out-of-sample regret of the resulting plan to 0.40% at 200 retained scenarios, against 11.91% and 0.88% for random subsampling, while performing comparably to fast-forward selection and plain medoid clustering. Decomposing the statistical error showed optimization error, at 11.59 $M, to exceed Monte Carlo sampling error at 7.99 $M and to dwarf the scenario-reduction bias of at most 0.80 $M. Under joint perturbation of six parameters, the value of lost load carried a standardized regression coefficient of 0.66 on total cost, ahead of crew capability at −0.53 and crew cost at 0.52, while the reserve scale itself proved weakly identified, ranging from 134.5 to 326.3 $M between the 5th and 95th percentiles. The cause mix dictated the resource portfolio, with disasters favouring crews and materials and cyber attacks favouring spare equipment, and the value of lost load was the dominant economic driver of the appropriate reserve scale.

The scope of these conclusions is bounded by the assumptions that generate them, and the principal limitations should be stated plainly. The study is a methodological proof of concept rather than a validated prescription: the instance is synthetic, the hazard models are not calibrated against historical outage records, and network operability is absent, so the numerical values characterize the structure of the preparedness trade-off rather than the appropriate reserve for any particular utility. The restoration model releases reusable resources at the end of every period and represents delay only through aggregate reachability, which omits travel time, service duration and resource occupancy and therefore describes an upper envelope on restoration speed. Resource movement is represented by aggregate availability rather than by origin–destination flows with depot balances, so the model supports conclusions about the scale and composition of the pre-positioned portfolio but not about detailed spatial allocation. The reserve is a single liquid pool priced by one holding rate, so catastrophe insurance and contingent credit, which differ in trigger, payout timing, coverage limit and counterparty risk, are not represented as distinct instruments. The scenario process assigns each scenario a single cause, and the compound-hazard analysis above shows that this understates the tail, modestly over the range tested but not negligibly. Social consequences are monetized through the value of lost load alone, so distributional effects across customer classes, the position of hospitals and other critical services beyond their higher unit valuation, regulatory reliability standards and equity in restoration priority are outside the present objective. Finally, the cyber class is represented at the level of its outage footprint rather than its cyber-physical mechanism, so the results concern a synthetic outage regime and not cyber resilience in general.

These limitations map directly onto the extensions that would most improve the framework. Representing the reserve as a portfolio of cash, parametric insurance and contingent credit, each with its own trigger, cost and basis risk, would turn the reserve-sizing question into an instrument-choice question and connect the model to the disaster risk finance literature [25]. Embedding network operability, so that restored load must be deliverable rather than merely energized, would sharpen the spatial allocation and permit island-level analysis; adding origin–destination dispatch with travel times and depot balances would do the same for the logistics layer [26]. Replacing the exclusive cause indicator with a joint cause model would let compound and cascading hazards be priced directly [36]. Calibrating hazard frequencies, footprints and restoration productivities against historical outage, weather and cyber-incident records would move the study from proof of concept towards empirical validation, and would also permit a distributional objective in which equity across customer classes is optimized alongside cost. On the technology side, the growing role of grid-scale and mobile battery storage as an emergency re-energization resource makes the state of health and remaining useful life of those assets a determinant of the capability that a reserve actually buys, since a mobile storage unit contributes to restoration only to the extent that its usable capacity and power rating are known and maintained; recent work on whole-life-cycle health and remaining-useful-life estimation for lithium-ion batteries [46], on multi-time-scale terminal-voltage characterization and retirement prediction for large-capacity batteries in energy storage stations [47], and on online co-estimation of state of charge and state of energy for large-scale storage [48] provides the estimation machinery that a storage-aware extension of the resource model would need. Finally, extending the two-stage formulation into a multi-stage decision that updates the reserve as threats evolve, and coordinating preparedness among neighbouring utilities, would broaden the framework towards system-wide resilience.
